# ERRα-KDM5C restrains STING enhancer activity to modulate type I interferon signaling in breast cancer progression

**DOI:** 10.1038/s41419-026-08499-2

**Published:** 2026-02-18

**Authors:** Zu-Hui Xu, Jie Chen, Ying He, Cheng Lei, Xiao-Ling Wang, De-Fa Huang, Zheng-Zhe Li, Hui Zhou, Mei-Yan Wang, Cheng-Gui Song, Juan Lin, Wen Liu, Xiao-Nan Wu, Wen-Juan Zhang

**Affiliations:** 1 Department of Laboratory Medicine, First Affiliated Hospital of Gannan Medical University, Ganzhou, Jiangxi China; 2 School of Medical Technology, Gannan Medical University, Ganzhou, Jiangxi China; 3 Department of Laboratory Medicine, The Affiliated Yongchuan Hospital of Chongqing Medical University, Chongqing, China; 4 Laboratory Animal Research Center, Xiamen University, Xiamen, Fujian China; 5 State Key Laboratory of Cellular Stress Biology, School of Pharmaceutical Sciences, Faculty of Medicine and Life Sciences, Xiamen University, Xiamen, Fujian China; 6 Fujian Provincial Key Laboratory of Innovative Drug Target Research, School of Pharmaceutical Sciences, Faculty of Medicine and Life Sciences, Xiamen University, Xiamen, Fujian China; 7 State Key Laboratory of Cellular Stress Biology, Cancer Research Center, School of Medicine, Faculty of Medicine and Life Sciences, Xiamen University, Xiamen, Fujian China; 8 The First School of Clinical Medicine, Gannan Medical University, Ganzhou, Jiangxi China; 9 Precision Medicine Center, First Affiliated Hospital of Gannan Medical University, Ganzhou, Jiangxi China; 10 Ganzhou Key Laboratory of Immunotherapeutic Drugs Developing for Childhood Leukemia, Gannan Medical University, Ganzhou, Jiangxi China

**Keywords:** Histone post-translational modifications, Breast cancer, Transcriptional regulatory elements, Innate immunity, Immune evasion

## Abstract

Regulation of enhancer activity plays a pivotal role in governing gene expression and cellular behaviors. However, the precise mechanisms underlying dynamic control of active enhancers remain incompletely defined. Here, we demonstrate that the nuclear receptor estrogen-related receptor α (ERRα) forms a functional complex with the H3K4me3-specific demethylase KDM5C to co-occupy a large set of active enhancers, including the locus of STING. In breast cancer cells, ERRα depletion induces STING enhancer hyperactivation, evidenced by H3K4me3 deposition, decreased H3K4me1, and increased enhancer RNA (eRNA) transcription. Accordingly, depletion of ERRα leads to further activation of STING gene transcription and TBK1-IRF3 pathway, accompanied by increased type I interferon (IFN) and IFN-stimulated gene (ISG) expression, as confirmed by transcriptomic analysis. Notably, depleting ERRα markedly attenuates breast tumor cell growth in vitro and in vivo, and our in vitro evidence indicates this occurs in part through activating STING signaling. These findings establish that the ERRα-KDM5C serves as a critical checkpoint for STING enhancer activity, revealing a regulatory mechanism of STING enhancer activity in breast tumor progression.

## Highlights

- ERRα interacts with KDM5C to co-occupy active enhancers, including the locus of STING.
- ERRα depletion induces STING enhancer hyperactivation through KDM5C displacement and H3K4me3 deposition.
- STING-TBK1-IRF3 signaling activation mediates enhanced type I IFN/ISG expression upon ERRα depletion.
- ERRα contributes to breast tumor cell growth in vitro and in vivo, and our in vitro evidence indicates this occurs in part through suppression of STING signaling.

## Introduction

Epigenetic reprogramming is an emerging hallmark of cancer [1, 2]. While enhancers are well-known genetic regulatory elements that control gene expression, the activity of enhancers is tightly controlled by chromatin-modifying enzymes and transcriptional regulators [3]. Dysregulation of enhancer activity has been widely documented in various types of cancers, generating aberrant gene regulatory state that drives the uncontrolled growth of cancer cells [4]. The functional states of enhancers are defined by distinct histone modification signatures: poised enhancers exhibit H3K4me1 with bivalent H3K27me3, primed enhancers display H3K4me1 alone, while active enhancers are characterized by H3K4me1/H3K27ac co-occupancy accompanied by MED1/p300/CBP recruitment [5–7]. This epigenetic signature enables enhancer-promoter looping and enhancer RNA (eRNA) transcription, which are critical for target gene activation [8, 9]. Notably, super-enhancers—large clusters of enhancers with exceptionally high levels of H3K27ac and MED1—have been identified as key drivers of oncogene expression in multiple cancers [10–13]. Within this framework, the methylation status of H3K4 dynamically correlates with enhancer potency. While H3K4me1 serves as a basal mark for primed enhancers, the deposition of H3K4me3 within active enhancers has been increasingly recognized to potentiate transcriptional output by chromatin accessibility [14–17].

The H3K4-specific demethylase KDM5C (also known as JARID1C or SMCX) [18, 19] embodies the contextual complexity of epigenetic regulation in cancer, exhibiting both tumor-suppressive and oncogenic functions in a cell-type and context-dependent manner [20]. As a putative tumor suppressor, KDM5C represses oncogenic programs by demethylating H3K4me2/3 at critical enhancers, as demonstrated in cervical [21–23], cholangiocarcinoma [24], clear cell renal carcinoma [25, 26], papillary thyroid carcinoma [27], breast cancer [28], and acute myeloid leukemia [29]. Conversely, a substantial body of evidence has uncovered oncogenic roles for KDM5C, where it promotes malignant phenotypes in prostate cancer [30–32], colon cancer [33], ovarian cancer [34], breast cancer [35], nasopharyngeal carcinoma [36], diffuse large B-cell lymphoma [37], bladder cancer [38] and glioblastoma multiforme [39]. These seemingly contradictory roles underscore the critical need to elucidate the precise molecular mechanisms and cellular contexts that determine KDM5C’s function. Emerging evidence suggests that KDM5C-mediated erasure of H3K4me3 may act as a molecular switch fine-tuning enhancer activity [17, 22, 28, 40, 41]. However, despite these advances, a significant gap remains in understanding how KDM5C interacts with key transcriptional regulators to modulate enhancer-driven oncogenic signaling pathways in specific cancers.

Recently, we reported that KDM5C promotes ERα-positive breast cancer growth [35]. In the current study, we reveal a novel mechanism that KDM5C contributes to breast cancer progression through estrogen-related receptor alpha (ERRα)-regulated enhancer activity of stimulator of interferon genes (STING) [42], which controls innate immune pathway and impacts tumorigenesis.

## Results

### KDM5C interacts with ERRα at enhancers

**Fig. 1:**
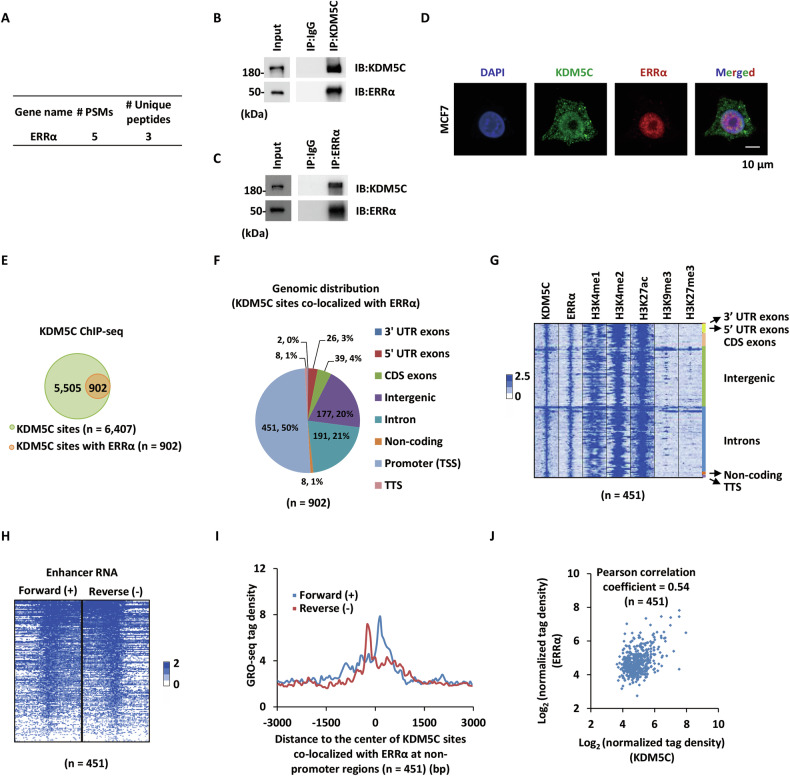
KDM5C interacts with ERRα at enhancers. **A** MCF7 cells stably expressing Flag-HA-tagged KDM5C were subjected to affinity purification and mass spectrometry (MS) analysis. The number of peptide spectrum matches (PSMs) and unique peptides for ERRα identified to interact with KDM5C was shown as indicated. **B** MCF7 cells were subjected to immunoprecipitation (IP) with anti-KDM5C antibody, followed by immunoblotting (IB) analysis as indicated. **C** MCF7 cells were subjected to IP with anti-ERRα antibody followed by IB analysis as indicated. **D** MCF7 cells were subjected to immunofluorescence analysis. Nuclei (DAPI), KDM5C (green), ERRα (red). Scale bars, 10 μm. **E** MCF7 cells were subjected to ChIP-seq with anti-KDM5C-specific antibody. KDM5C binding sites that overlapped with ERRα were shown as indicated. **F** Genomic distribution of KDM5C and ERRα co-bound sites (*n* = 902) is shown by pie chart. **G** Heat map representation of KDM5C, ERRα, H3K4me1, H3K4me2, H3K27ac, H3K9me3, and H3K27me3 ChIP-seq tag density centered on KDM5C sites that co-localized with ERRα (±3000 bp). eRNA levels on both sense (+) and antisense (−) strands on KDM5C and ERRα co-bound sites detected by GRO-seq are shown by heat map (**H**) and tag density plot (**I**) (±3000 bp). **J** The correlation between the ChIP-seq tag density of KDM5C and ERRα on co-bound enhancers (±1000 bp).

To elucidate the mechanistic role of KDM5C in breast cancer progression, we performed affinity purification coupled mass spectrometry (MS) to identify its interacting partners. MS results identified orphan nuclear receptor ERRα (Fig. 1A), which shares high homology with estrogen receptor α (ERα) in DNA binding domain (DBD) [43]. We demonstrated the interaction between KDM5C and ERRα at both endogenous (Fig. 1B, C) and exogenous levels (Fig. S1A). We then mapped the domain in KDM5C that interacts with ERRα by co-expressing full-length or different truncated forms of KDM5C with ERRα, followed by immunoprecipitation (IP). The interaction region was first determined to be the amino (N)-terminal of KDM5C, encompassing the JmjN, BRIGHT, PHD, and JmjC domains (Fig. S1B). Confocal immunofluorescence (IF) demonstrated decent nuclear co-localization of KDM5C and ERRα in MCF7 cells (Fig. 1D).

The interaction prompted us to examine whether ERRα and KDM5C co-localize on chromatin. The chromatin immunoprecipitation followed by sequencing (ChIP-seq) data for KDM5C and ERRα were reanalyzed from publicly available datasets, including our previous study [35] and ENCODE dataset (GSM2424192). Profiling of ERRα and KDM5C chromatin binding sites by ChIP-seq revealed that approximately 14% of KDM5C binding sites were co-occupied by ERRα (*n* = 902) (Fig. 1E). ERRα and KDM5C co-bound sites were predominantly located in promoter-proximal and distal non-promoter regions (Fig. 1F). Strikingly, these non-promoter loci exhibited active enhancer hallmarks, characterized by enriched H3K4me1/2 and H3K27ac signals, but devoid of repressive H3K9me3 or H3K27me3 marks (Fig. 1G). Consistent with enhancer activity, GRO-seq analysis revealed bidirectional enhancer RNA (eRNA) transcription at co-bound sites (Fig. 1H), with tag density peaks overlapping ChIP-seq signals of ERRα and KDM5C (Fig. 1I). Quantitative correlation analysis demonstrated strong correlation of ERRα and KDM5C ChIP-seq signals at co-bound enhancers (Fig. 1J). Taken together, these data suggested that KDM5C interacts with ERRα, and they are largely co-localized on active enhancer regions.

### ERRα and KDM5C repress type I IFN-stimulated gene expression

**Fig. 2:**
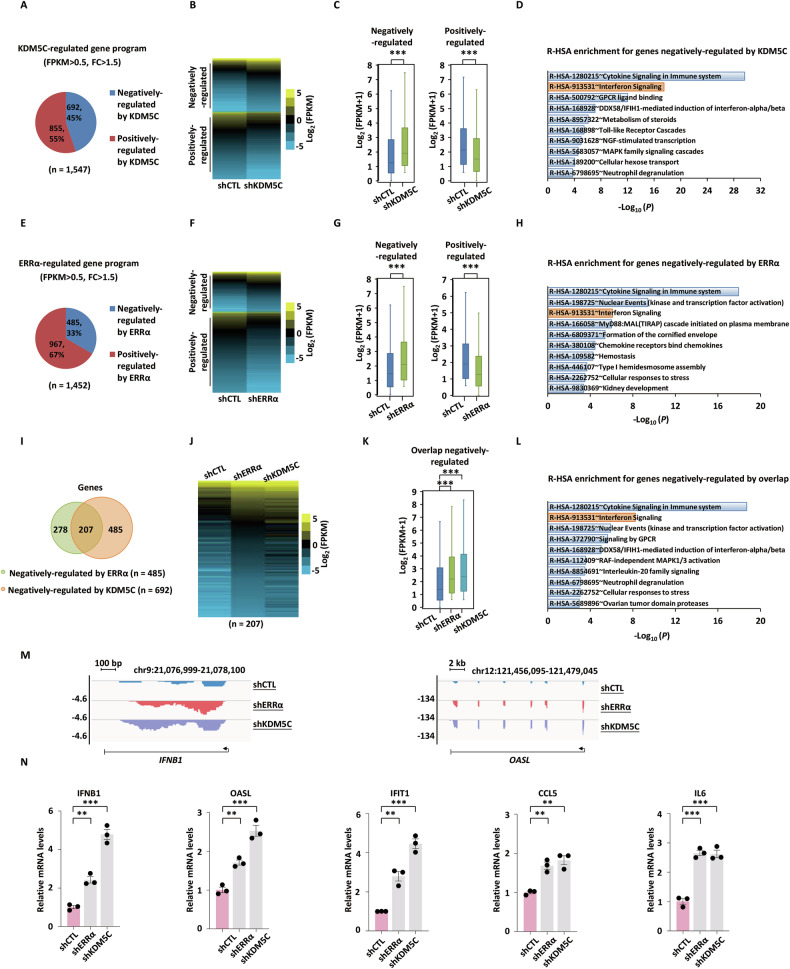
ERRα and KDM5C repress type I IFN-stimulated gene expression. **A** MCF7 cells were infected with a non-targeting control shRNA (shCTL) or a shRNA specifically targeting KDM5C (shKDM5C), followed by RNA-seq analysis. Genes positively and negatively regulated by KDM5C are shown by a pie chart. Heat map (**B**) and box plot (**C**) representation of the expression (log_2_(FPKM + 1)) for genes regulated by KDM5C (unpaired Student’s *t*-test, two-tailed). **D** Reactome pathway for Homo sapiens (R-HSA) enrichment analysis for genes negatively regulated by KDM5C. Top 10 enriched Reactome pathways are shown. **E** MCF7 cells were infected with shCTL or shERRα followed by RNA-seq analysis. Genes positively and negatively regulated by ERRα are shown by a pie chart. Heat map (**F**) and box plot (**G**) representation of the expression (log_2_(FPKM + 1)) for genes regulated by ERRα. **H** R-HSA enrichment analysis for genes negatively regulated by ERRα. Top 10 enriched Reactome pathways are shown. Heat map (**J**) and box plot (**K**) representation of the expression (log_2_(FPKM + 1)) for genes negatively regulated by both KDM5C and ERRα (**I**) (unpaired Student’s *t*-test, two-tailed). **L** R-HSA enrichment analysis for genes negatively regulated by both KDM5C and ERRα. Top 10 enriched Reactome pathways are shown. **M** Genome browser views of canonical type I IFNs and IFN-stimulated genes (ISGs) such as IFNB1 and OASL based on RNA-seq are shown using the Integrative Genomics Viewer (IGV). Blue, shCTL; red, shERRα; purple, shKDM5C. **N** MCF7 cells infected with shCTL, shERRα, or shKDM5C were subjected to RT-qPCR analysis, and the expression of representative genes such as IFNB1, OASL, IFIT1, CCL5, and IL6 is shown. The experiments were repeated three times, and the representative data are shown (mean ± SEM; *n* = 3; *P < 0.05, **P < 0.01, ***P < 0.001; unpaired Student’s *t-*test, two-tailed).

To elucidate the transcriptional regulatory roles of KDM5C and ERRα, we performed RNA sequencing (RNA-seq) to compare gene expression profiles in MCF7 cells infected with shRNAs targeting KDM5C (shKDM5C) or ERRα (shERRα) versus a non-targeting control shRNA (shCTL). Visualization of the sequencing data confirmed that both shRNAs efficiently reduced the mRNA levels of their respective target genes (Fig. S2A, B). Notably, knockdown of ERRα did not significantly alter KDM5C expression, nor did KDM5C depletion affect ERRα mRNA levels, indicating the specificity of the knockdown effects (Fig. S2A, B). Differential expression analysis identified 855 and 692 genes positively and negatively regulated by KDM5C, respectively (fragments per kilobase of transcript per million mapped reads, FPKM > 0.5, fold change > 1.5) (Fig. 2A). The expression patterns of these KDM5C-regulated genes were shown by heat map (Fig. 2B) and box plot (Fig. 2C). To gain insights into the biological functions and signaling pathways KDM5C is involved in, functional enrichment analysis of reactome pathway gene sets was performed. IFN signaling pathway (R-HSA:913531, *P* = 3.2 × 10^−^^18^) was found to be one of the most significantly enriched pathways for genes negatively regulated by KDM5C (Fig. 2D). Similarly, differential expression analysis identified 967 and 485 genes positively and negatively regulated by ERRα, respectively (FPKM > 0.5, fold change > 1.5) (Fig. 2E). The expression patterns of these ERRα-regulated genes were shown by heat map (Fig. 2F) and box plot (Fig. 2G). Intriguingly, reactome pathway enrichment analysis revealed that genes negatively regulated by ERRα were also significantly enriched in IFN signaling pathway (R-HSA:913531, *P* = 8.3 × 10⁻^7^) (Fig. 2H). Additionally, we also performed single-sample gene set enrichment analysis (ssGSEA), a method specifically designed for calculating enrichment scores in individual samples. The results confirmed the significant enrichment of interferon response (Fig. S2C) and type I IFN signaling pathway (Fig. S2D), aligning perfectly with Metascape findings.

Furthermore, the KDM5C and ERRα co-repressed gene sets (Fig. 2I) were confirmed by heat map (Fig. 2J) and box plot (Fig. 2K). Reactome pathway enrichment analysis revealed that genes negatively regulated by KDM5C and ERRα were also significantly enriched in type I IFN signaling pathway (R-HSA:913531, *P* = 5.8 × 10⁻^9^) (Fig. 2L). Notably, canonical type I IFNs and IFN-stimulated genes (ISGs), such as IFNB1 and OASL, exhibited marked upregulation in both shKDM5C and shERRα conditions (Fig. 2M). Moreover, RT-qPCR validation further demonstrated that silencing KDM5C or ERRα significantly increased expression of type I IFNs and ISGs, such as IFNB1, OASL, IFIT1, CCL5, and IL6 (Fig. 2N). Taken together, our data suggested that ERRα and KDM5C repress type I IFN-stimulated gene expression in breast cancer cells.

### ERRα and KDM5C depletion activates STING gene transcription

**Fig. 3:**
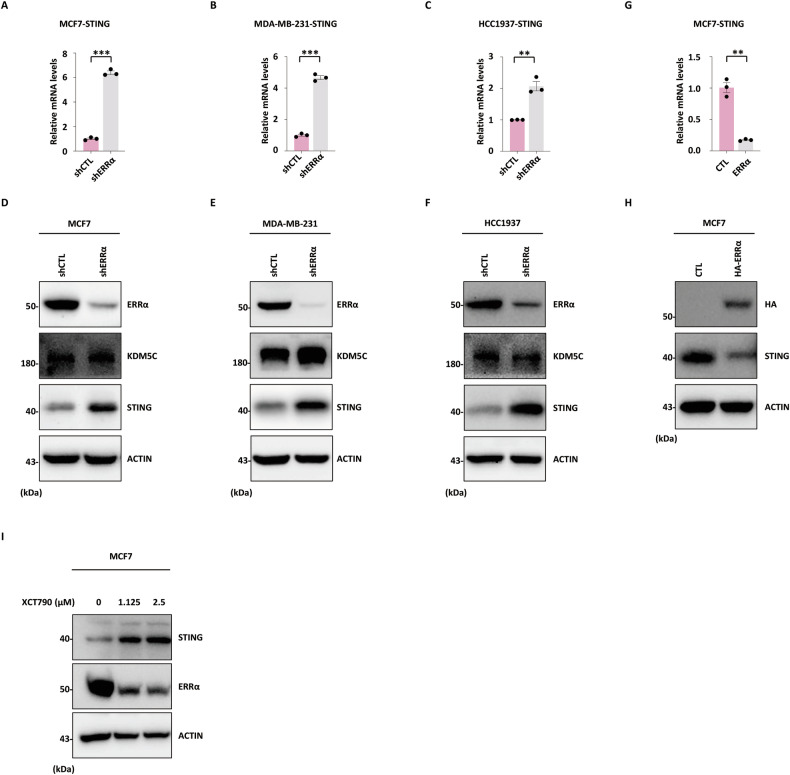
ERRα and KDM5C depletion relieves STING transcriptional repression. MCF7 (**A**), MDA-MB-231 (**B**), and HCC1937 (**C**) cells infected with shCTL or shERRα were subjected to RT-qPCR analysis, and the expression of STING is shown. The experiments were repeated three times, and the representative data are shown (mean ± SEM; *n* = 3; **P < 0.01, ***P < 0.001; unpaired Student’s *t-*test, two-tailed). MCF7 (**D**), MDA-MB-231 (**E**), and HCC1937 (**F**) cells described in (**A**–**C**) were subjected to IB analysis using antibodies as indicated. Molecular weight is indicated on the left (in kDa). **G** MCF7 cells infected with a control vector or an ERRα-expressing vector were subjected to RT-qPCR analysis, and the expression of STING is shown. The experiments were repeated three times, and the representative data are shown (mean ± SEM; *n* = 3; **P < 0.01; unpaired Student’s *t-*test, two-tailed). **H** MCF7 cells described in (**G**) were subjected to IB analysis using antibodies as indicated. Molecular weight is indicated on the left (in kDa). **I** MCF7 cells treated with XCT790 for 48 h were subjected to IB analysis using antibodies as indicated. Molecular weight is indicated on the left (in kDa).

Previous studies have established that KDM5C suppresses type I IFN signaling by inhibiting the transcription of STING [44], a critical adapter protein mediating TBK1-IRF3 signaling in the IFN pathway [42]. To investigate the transcriptional regulation of STING by ERRα, we performed RT-qPCR in three breast cancer cell lines, including MCF7, MDA-MB-231, and HCC1937, transduced with shRNA targeting ERRα (shERRα) or a non-targeting control shRNA (shCTL). ERRα knockdown significantly upregulated STING mRNA levels in all these cell lines (Fig. 3A–C). Consistent with the mRNA data, immunoblot analysis confirmed that ERRα depletion significantly increased STING protein levels, while KDM5C protein expression remained unchanged (Fig. 3D–F).

To validate the causal role of ERRα in STING repression, we performed gain-of-function experiments. Ectopic expression of ERRα potently suppressed both STING mRNA (Fig. 3G) and protein levels (Fig. 3H). Furthermore, treatment with XCT790, a selective ERRα inverse agonist, potently induced STING protein levels in a dose-dependent manner (Fig. 3I). These reciprocal data demonstrate that ERRα functions as a transcriptional repressor of STING in breast cancer cells.

We next asked whether KDM5C knockdown produces a similar effect on STING expression. Indeed, in line with the observations following ERRα depletion, KDM5C knockdown also significantly increased STING expression in MCF7, MDA-MB-231, and HCC1937 cells at both mRNA (Fig. S3A–C) and protein levels (Fig. S3D–F). Importantly, ERRα protein levels were not altered under these conditions (Fig. S3D–F). Collectively, these data suggested that KDM5C and ERRα may function in a convergent or cooperative manner to repress STING transcription.

To examine whether the ERRα-KDM5C complex regulates other key innate immune sensors, we analyzed the expression of cGAS, MDA5, and MAVS following knockdown of ERRα or KDM5C. Our RNA-seq data revealed that knockdown of either gene significantly increased MDA5 mRNA levels, whereas the mRNA levels of cGAS and MAVS remained unchanged (Fig. S4A–C). These findings suggest a specific regulatory effect on MDA5. However, ChIP-seq data indicated that ERRα, but not KDM5C, binds to the promoter region of MDA5 (Fig. S4B), hinting that they may regulate MDA5 through distinct mechanisms. In fact, our recent independent study confirmed that ERRα directly binds to the promoters of RIG-I and MDA5 to suppress their expression [45]. Crucially, among the sensors analyzed, STING emerged as the only convergent target co-regulated by both ERRα and KDM5C at the enhancer level, underscoring its central and specific role in the mechanism identified in our study.

### ERRα and KDM5C depletion increases STING enhancer activity

**Fig. 4:**
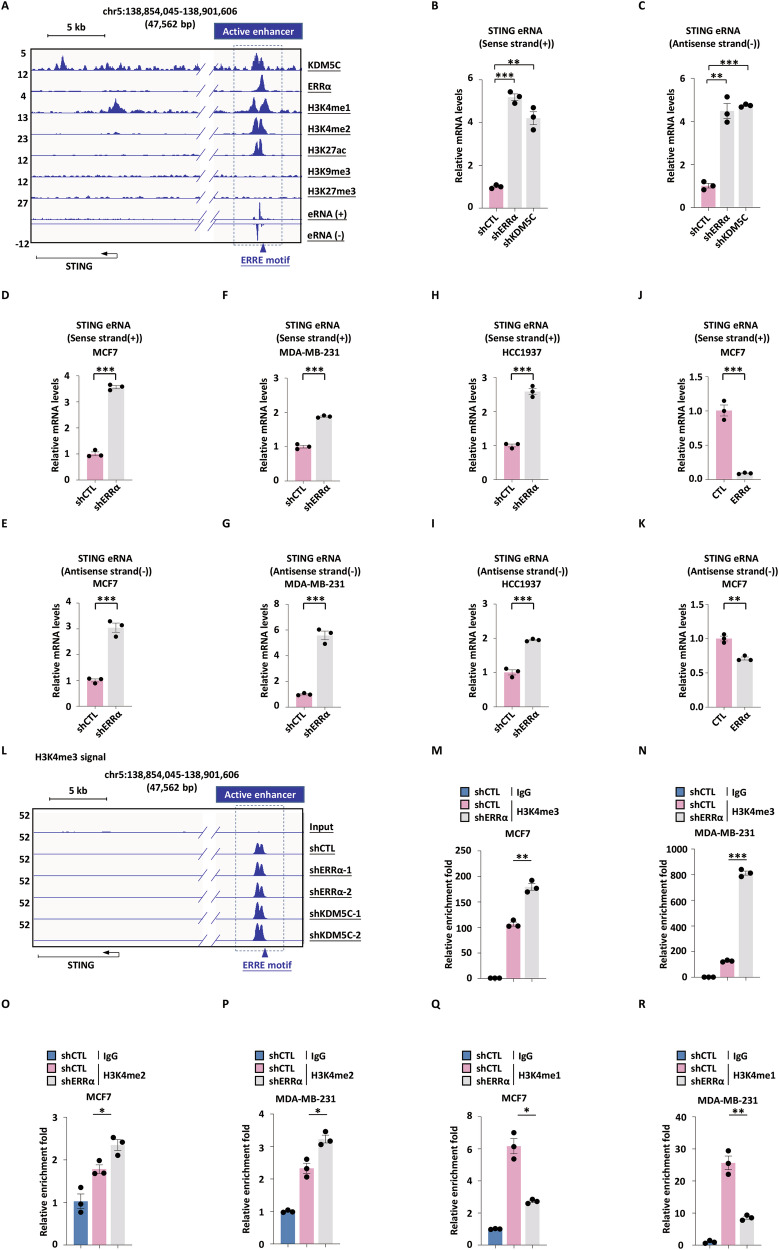
ERRα and KDM5C depletion increases STING enhancer activity. **A** Genomic views of KDM5C, ERRα, H3K4me1, H3K4me2, H3K27ac, H3K9me3, and H3K27me3 ChIP-seq and GRO-seq (eRNAs) tracks on the enhancer region in the vicinity of STING gene. The boxed region indicates STING enhancer. MCF7 cells infected with shCTL, shERRα, or shKDM5C were subjected to RT-qPCR analysis, and the expression of sense (+) (**B**) and antisense (−) (**C**) strands of STING eRNA is shown. The experiments were repeated three times, and the representative data are shown (mean ± SEM; *n* = 3; **P < 0.01; ***P < 0.001; unpaired Student’s *t-*test, two-tailed). MCF7 (**D**, **E**), MDA-MB-231 (**F**, **G**), and HCC1937 cells (**H**, **I**) infected with shCTL or shERRα were subjected to RT-qPCR analysis, and the expression of sense (+) and antisense (−) strands of STING eRNA is shown. The experiments were repeated three times, and the representative data are shown (mean ± SEM; *n* = 3; ***P < 0.001; unpaired Student’s *t-*test, two-tailed). MCF7 cells infected with a control vector or an ERRα-expressing vector were subjected to RT-qPCR analysis, and the expression of sense (+) (**J**) and antisense (−) (**K**) strands of STING eRNA is shown. The experiments were repeated three times, and the representative data are shown (mean ± SEM; *n* = 3; **P < 0.01, ***P < 0.001; unpaired Student’s *t-*test, two-tailed). **L** Genomic views of H3K4me3 on the enhancer region in the vicinity of STING gene in MCF7 cells infected with shCTL, shERRα, or shKDM5C. The boxed region indicates STING enhancer. MCF7 (**M**) and MDA-MB-231 cells (**N**) infected with shCTL or shERRα were subjected to CUT&Tag with normal IgG or anti-H3K4me3 antibody and qPCR with primers specifically targeting enhancer regions of STING gene. CUT&Tag signals are presented as relative enrichment fold. The experiments were repeated three times, and the representative data are shown (mean ± SEM; *n* = 3; **P < 0.01, ***P < 0.001; unpaired Student’s *t-*test, two-tailed). MCF7 (**O**) and MDA-MB-231 cells (**P**) infected with shCTL or shERRα were subjected to CUT&Tag with normal IgG or anti-H3K4me2 antibody and qPCR with primers specifically targeting enhancer regions of STING gene. CUT&Tag signals are presented as relative enrichment fold. The experiments were repeated three times, and the representative data are shown (mean ± SEM; *n* = 3; *P < 0.05; unpaired Student’s *t-*test, two-tailed). MCF7 (**Q**) and MDA-MB-231 cells (**R**) infected with shCTL or shERRα were subjected to ChIP with normal IgG or anti-H3K4me1 antibody and qPCR with primers specifically targeting enhancer regions of STING gene. ChIP signals are presented as relative enrichment fold. The experiments were repeated three times, and the representative data are shown (mean ± SEM; *n* = 3; *P < 0.05, **P < 0.01; unpaired Student’s *t-*test, two-tailed).

Intriguingly, we found that ERRα and KDM5C co-localize at the STING enhancer characterized by enriched H3K4me1/2 and H3K27ac signals, but devoid of repressive H3K9me3 or H3K27me3 marks, with bidirectional eRNA transcription (Fig. 4A). Knockdown of ERRα or KDM5C led to significantly increased sense and antisense eRNA transcription of STING in MCF7 cells (Fig. 4B, C). To further assess the functional role of ERRα in STING enhancer regulation, we performed RT-qPCR in MCF7 (Fig. 4D, E), MDA-MB-231 (Fig. 4F, G), and HCC1937 (Fig. 4H, I) breast cancer cells transduced with shERRα or shCTL. ERRα knockdown markedly increased both sense and antisense eRNA transcription at the STING enhancer in all three cell lines (Fig. 4D–I). To validate the causal relationship, we conducted gain-of-function assays. Ectopic expression of ERRα significantly suppressed both sense and antisense STING eRNA transcription (Fig. 4J, K). These data establish ERRα as a transcriptional repressor of STING enhancer activity in breast cancer cells.

To directly test whether the enzymatic activity is required, we performed an additional experiment. As shown in Fig. S5A, B, exogenous expression of wild-type KDM5C in MCF7 cells successfully suppressed STING enhancer activation, as measured by enhancer RNA levels. In contrast, expression of a catalytically inactive mutant (H514A) not only failed to repress but further increased STING eRNA levels, suggesting a potential dominant-negative effect. Consistent with this, we observed similar regulation at the STING protein level (Fig. S5C). These results provide direct evidence that the demethylase activity of KDM5C is essential for its repressive function at the STING enhancer. Western blot analysis confirmed that knockdown of either KDM5C or ERRα resulted in a significant accumulation of H3K4me2 and H3K4me3, alongside a slight increase in H3K4me1, at the cellular level in MCF7 cells (Fig. S5D, E).

Emerging evidence suggests that KDM5C-mediated H3K4me3 regulation represents a molecular switch governing enhancer activity [17, 22, 28, 40, 41]. To investigate the genomic function of the ERRα-KDM5C complex, we performed H3K4me3 ChIP-seq in MCF7 cells expressing a non-targeting control shRNA (shCTL) or one of two independent shRNAs targeting KDM5C (shKDM5C-1, shKDM5C-2) or ERRα (shERRα-1, shERRα-2). The results showed that knockdown of either KDM5C or ERRα increased H3K4me3 levels at their co-occupied genomic sites (Fig. S5F–H), with a pronounced increase at the STING enhancer region (Fig. 4L). We next employed cleavage under targets and tagmentation (CUT&Tag) and conventional ChIP to assess changes in H3K4me3, H3K4me2, and H3K4me1 marks following ERRα depletion. Depletion of ERRα markedly elevated H3K4me3 levels at the STING enhancer in both MCF7 (Fig. 4M) and MDA-MB-231 cells (Fig. 4N). Similarly, H3K4me2 levels were elevated at this enhancer in MCF7 (Fig. 4O) and MDA-MB-231 (Fig. 4P) cells. In contrast, H3K4me1 levels were decreased upon ERRα knockdown in MCF7 (Fig. 4Q) and MDA-MB-231 cells (Fig. 4R). Collectively, these findings indicate that ERRα-KDM5C-mediated H3K4me3 demethylation contributes to the transcriptional suppression of the STING enhancer in breast cancer cells.

### ERRα restrains STING-TBK1-IRF3 signaling in breast cancer cells

**Fig. 5:**
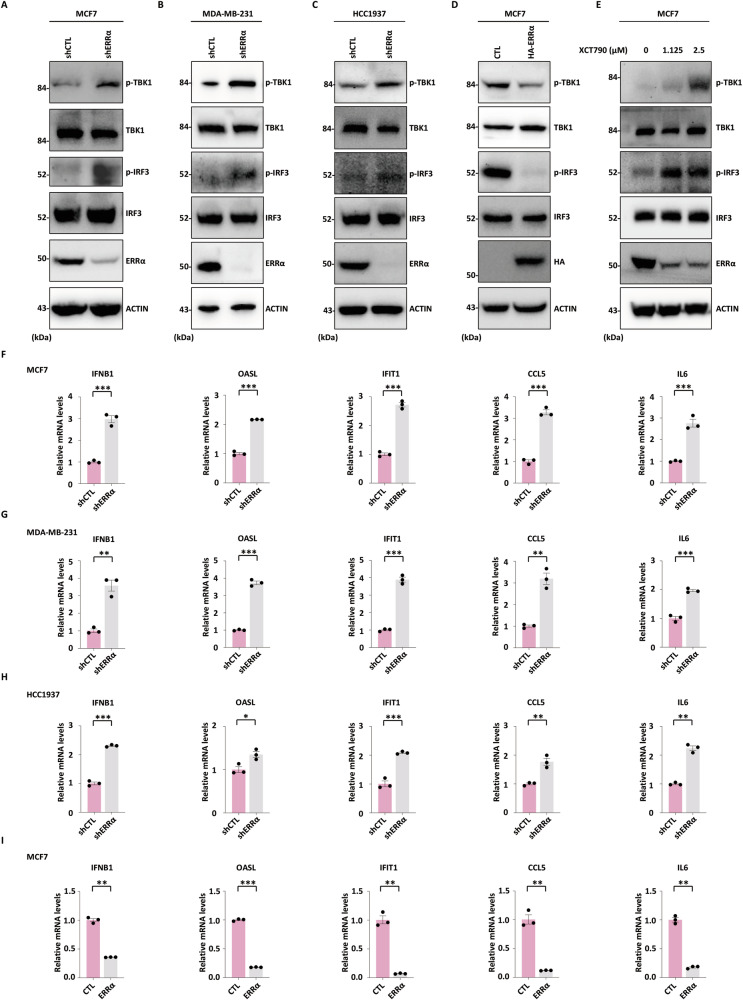
ERRα restrains STING-TBK1-IRF3 signaling in breast cancer cells. MCF7 (**A**), MDA-MB-231 (**B**), and HCC1937 (**C**) cells infected with shCTL or shERRα were subjected to IB analysis using antibodies as indicated. Molecular weight is indicated on the left (in kDa). **D** MCF7 cells infected with a control vector or an ERRα-expressing vector were subjected to IB analysis using antibodies as indicated. Molecular weight is indicated on the left (in kDa). **E** MCF7 cells treated with XCT790 as indicated for 48 h were subjected to IB analysis using antibodies as indicated. Molecular weight is indicated on the left (in kDa). MCF7 (**F**), MDA-MB-231 (**G**), and HCC1937 cells (**H**) infected with shCTL or shERRα were subjected to RT-qPCR analysis, and the expression of type I IFNs and ISGs such as IFNB1, OASL, IFIT1, CCL5, and IL6 is shown. The experiments were repeated three times, and the representative data are shown (mean ± SEM; *n* = 3; *P < 0.05, **P < 0.01, ***P < 0.001; unpaired Student’s *t-*test, two-tailed). **I** MCF7 cells infected with a control vector or an ERRα-expressing vector were subjected to RT-qPCR analysis, and the expression of type I IFNs and ISGs such as IFNB1, OASL, IFIT1, CCL5, and IL6 is shown. The experiments were repeated three times, and the representative data are shown (mean ± SEM; *n* = 3; **P < 0.01, ***P < 0.001; unpaired Student’s *t-*test, two-tailed).

To investigate whether ERRα modulates STING-mediated antiviral signaling, we examined phosphorylation status of TBK1 and IRF3, key downstream effectors in the STING pathway, following genetic or pharmacological perturbation of ERRα. Knockdown of ERRα markedly increased the phosphorylation of TBK1 and IRF3 in MCF7 (Fig. 5A), MDA-MB-231 (Fig. 5B), and HCC1937 cells (Fig. 5C), without altering their total protein levels. Conversely, ectopic expression of ERRα suppressed TBK1 and IRF3 phosphorylation (Fig. 5D), while total protein levels remained unchanged. Similarly, treatment with the ERRα inverse agonist XCT790 significantly increased phospho-TBK1 and phospho-IRF3 levels in MCF7 (Fig. 5E), again without affecting total protein expression.

We next assessed the functional output of this signaling activation by measuring the expression of type I IFN and ISGs. Functional downstream consequences were evaluated by RT-qPCR. ERRα depletion significantly upregulated IFNB1 and ISGs including OASL, IFIT1, CCL5, and IL6 in MCF7 (Fig. 5F), MDA-MB-231 (Fig. 5G), and HCC1937 breast cancer cells (Fig. 5H). Consistently, overexpression of ERRα led to repression of IFNB1 and ISGs (Fig. 5I). Taken together, these data demonstrate that ERRα acts as a negative regulator of the STING-TBK1-IRF3 signaling axis and subsequent type I IFN/ISG response in breast cancer cells.

### ERRα and KDM5C depletion suppresses breast cancer cell growth via STING

**Fig. 6:**
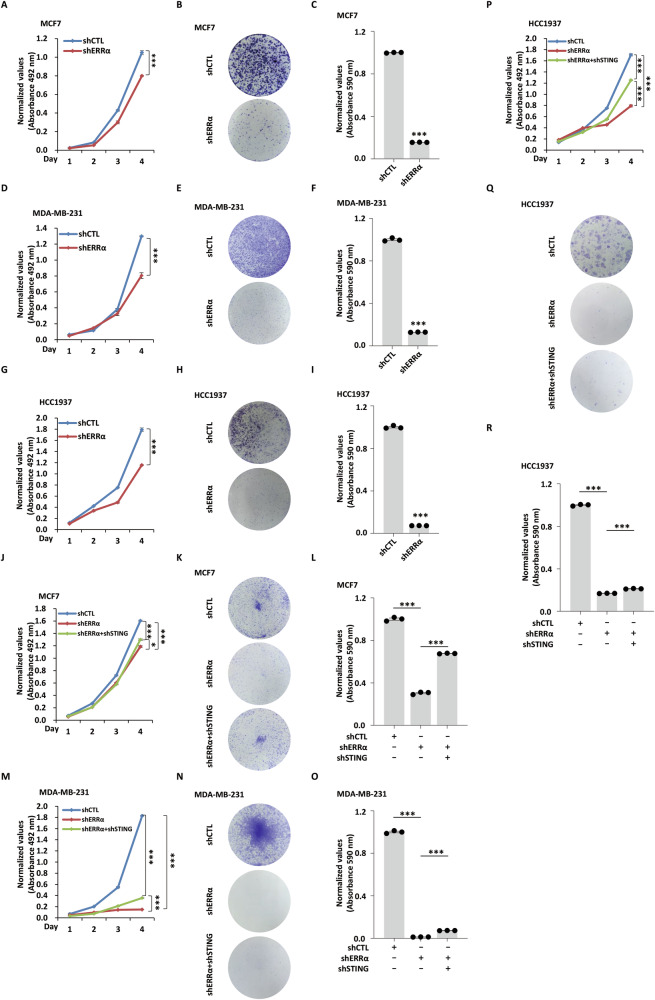
ERRα and KDM5C depletion suppresses breast cancer cell growth via STING. **A** MCF7 cells were infected with shCTL or shERRα, followed by cell proliferation assay (*n* = 3; mean ± SEM; ***P < 0.001; day 4, by unpaired two-tailed Student’s *t-*test). **B** MCF7 cells were infected with shCTL or shERRα, followed by colony formation assay. **C** The quantification of the crystal violet dye in (**B**) is shown (*n* = 3; mean ± SEM; ***P < 0.001). **D** MDA-MB-231 cells were infected with shCTL or shERRα, followed by cell proliferation assay (*n* = 3; mean ± SEM; ***P < 0.001; day 4, by unpaired two-tailed Student’s *t-*test). **E** MDA-MB-231 cells were infected with shCTL or shERRα, followed by colony formation assay. **F** The quantification of the crystal violet dye in (**E**) is shown (*n* = 3; mean ± SEM; ***P < 0.001). **G** HCC1937 cells were infected with shCTL or shERRα, followed by cell proliferation assay (*n* = 3; mean ± SEM; ***P < 0.001; day 4, by unpaired two-tailed Student’s *t-*test). **H** HCC1937 cells were infected with shCTL or shERRα, followed by colony formation assay. **I** The quantification of the crystal violet dye in (**H**) is shown (*n* = 3; mean ± SEM; ***P < 0.001). **J** MCF7 cells were infected with shCTL or shERRα together with or without shSTING, followed by cell proliferation assay (*n* = 3; mean ± SEM; *P < 0.05, ***P < 0.001; day 4, by unpaired two-tailed Student’s *t-*test). **K** MCF7 cells were infected with shCTL or shERRα together with or without shSTING, followed by colony formation assay. **L** The quantification of the crystal violet dye in (**K**) is shown (*n* = 3; mean ± SEM; ***P < 0.001). **M** MDA-MB-231 cells were infected with shCTL or shERRα together with or without shSTING, followed by cell proliferation assay (*n* = 3; mean ± SEM; ***P < 0.001; day 4, by unpaired two-tailed Student’s *t-*test). **N** MDA-MB-231 cells were infected with shCTL or shERRα together with or without shSTING, followed by colony formation assay. **O** The quantification of the crystal violet dye in (**N**) is shown (*n* = 3; mean ± SEM; ***P < 0.001). **P** HCC1937 cells were infected with shCTL or shERRα together with or without shSTING, followed by cell proliferation assay (*n* = 3; mean ± SEM; ***P < 0.001; day 4, by unpaired two-tailed Student’s *t-*test). **Q** HCC1937 cells were infected with shCTL or shERRα together with or without shSTING, followed by colony formation assay. **R** The quantification of the crystal violet dye in (**Q**) is shown (*n* = 3; mean ± SEM; **P < 0.01).

Given the established role of ERRα in suppressing type I IFN responses, we tested whether ERRα regulates breast cancer cell growth through this pathway. ERRα depletion markedly suppressed proliferation and colony formation in MCF7 (Fig. 6A–C), MDA-MB-231 (Fig. 6D–F), and HCC1937 (Fig. 6G–I) breast cancer cells. This finding aligns with prior evidence of ERRα‘s tumor-promoting role in breast cancer [45, 46]. Mechanistic validation supported that the proliferative rescue observed in ERRα-deficient cells depended on STING. Co-depletion of STING (shSTING) partially reversed ERRα knockdown-induced growth inhibition (Fig. 6J–L). Quantitative analysis revealed that dual knockdown (ERRα + STING) significantly restored proliferation rates in MCF7 cells (Fig. 6J–L), with similar trends in MDA-MB-231 (Fig. 6M–O) and HCC1937 (Fig. 6P–R) cells. These data demonstrated that ERRα promotes the malignant growth of breast cancer cells in part through STING.

We then examined whether KDM5C similarly regulates cell growth via type I IFN signaling. Indeed, in KDM5C-deficient cells, STING co-depletion rescued the growth inhibition in MCF7 (Fig. S6A–C), MDA-MB-231 (Fig.e S6D–F), and HCC1937 cell lines (Fig. S6G–I). This functional rescue mirrors the effect observed with ERRα, supporting a model in which both factors converge on STING to sustain proliferation in breast cancer cells.

### ERRα depletion increases antitumor immunity and efficacy of chemotherapy in breast tumors

**Fig. 7:**
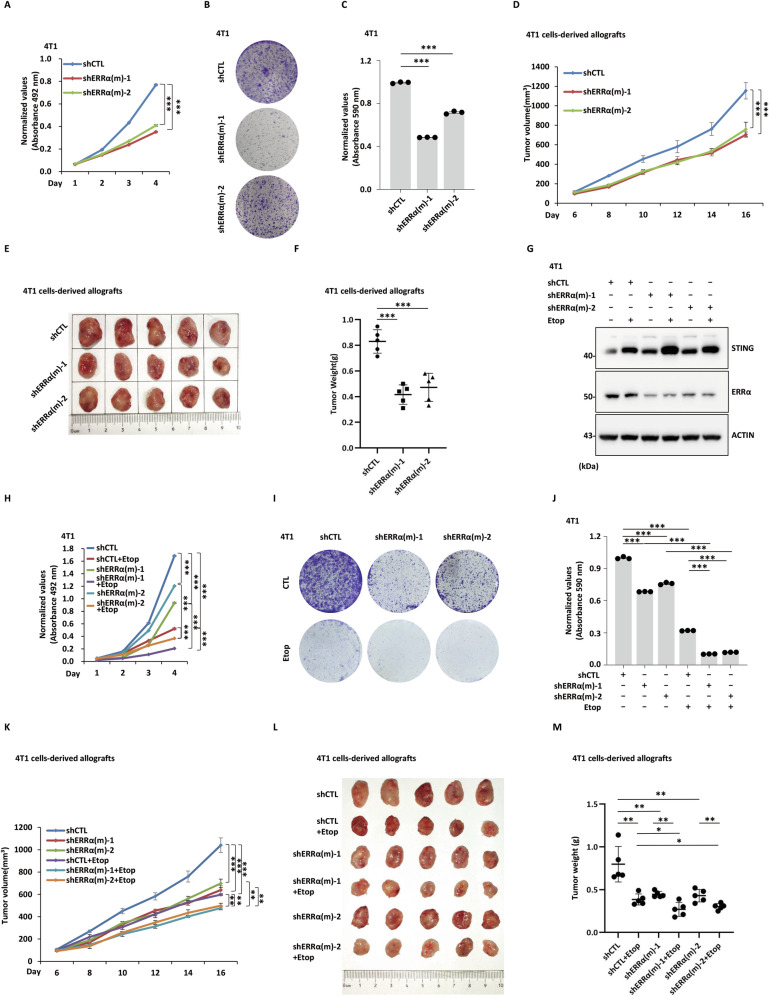
ERRα depletion inhibits breast tumor growth and increases the efficacy of chemotherapy. **A** 4T1 cells were infected with shCTL or two individual shERRα targeting mouse ERRα (shERRα(m)-1 and shERRα(m)-2), followed by cell proliferation assay (*n* = 3; mean ± SEM; ***P < 0.001; day 4, by unpaired two-tailed Student’s *t-*test). **B** 4T1 cells were infected with shCTL or shERRα(m)-1/2, followed by colony formation assay. **C** The quantification of the crystal violet dye in (**B**) is shown (*n* = 3; mean ± SEM; ***P < 0.001). **D** Female BALB/c mice were injected with shCTL or shERRα(m)-1/2 lentivirus-infected 4T1 cells. Tumor growth curve is shown (*n* = 5; mean ± SEM; ***P < 0.001). Tumors from the mice described in (**D**) were excised, and tumors were photographed (**E**) and weighted (**F**) (*n* = 5; mean ± SEM; ***P < 0.001). **G** 4T1 cells were infected with shCTL or two individual shERRα (shERRα(m)-1 and shERRα(m)-2) for 72 h before treating with or without etoposide (Etop, 50 μM) for 24 h, followed by IB analysis using antibodies as indicated. **H** 4T1 cells were infected with shCTL or two individual shERRα (shERRα(m)-1 and shERRα(m)-2) and then treated with or without etoposide (5 μM), followed by cell proliferation assay (*n* = 3; mean ± SEM; ***P < 0.001; day 4, by unpaired two-tailed Student’s *t-*test). **I** 4T1 cells were infected with shCTL or two individual shERRα (shERRα(m)-1 and shERRα(m)-2) and then treated with or without etoposide (5 μM), followed by colony formation assay. **J** The quantification of the crystal violet dye in (**I**) is shown (*n* = 3; mean ± SEM; ***P < 0.001). **K** Female BALB/c mice were injected with shCTL or shERRα(m) lentivirus-infected 4T1 cells and then treated with etoposide (8 mg/kg i.p.) every 2 days for 6 times. Tumor growth curve is shown (*n* = 5; mean ± SEM; **P < 0.01, ***P < 0.001; day 16, by unpaired two-tailed Student’s *t-*test). Tumors from the mice described in (**K**) were excised, photographed (**L**) and weighted (**M**) (*n* = 5; mean ± SEM; *P < 0.05, **P < 0.01; unpaired two-tailed Student’s *t-*test).

The critical role of ERRα in suppressing type I IFN responses led us to investigate whether ERRα regulates breast tumor cell growth. Knockdown of ERRα significantly suppressed the proliferation (Fig. 7A) and colony formation of 4T1 murine breast cancer cells (Fig. 7B, C). To further examine its in vivo relevance, we subcutaneously injected mice with 4T1 cells expressing a non-targeting control shRNA (shCTL) or two independent mouse shRNAs targeting ERRα (shERRα(m)-1/2). Consistent with the in vitro findings, knockdown of ERRα markedly inhibited tumor growth, reducing both tumor volume and tumor weight in BALB/c mice bearing 4T1 allografts (Fig. 7D–F).

We next investigated whether inhibiting ERRα enhances the efficacy of chemotherapy in suppressing breast tumor growth. To this end, we knocked down ERRα in 4T1 cells and treated them with etoposide (Fig. 7G), a chemotherapy agent that induces DNA damage and activates STING-mediated type I IFN responses. Notably, etoposide-induced STING expression was further elevated upon ERRα knockdown, indicating that the combination of ERRα inhibition and etoposide treatment exerts synergistic effects (Fig. 7G). Consistently, ERRα knockdown further potentiated the cytotoxicity of etoposide (Fig. 7H–J). We then extended these findings to an in vivo setting by subcutaneously injecting mice with 4T1 cells expressing a non-targeting control shRNA (shCTL) or two independent mouse shRNAs targeting ERRα (shERRα(m)-1/2), followed by treatment with etoposide or vehicle control. Aligning with the in vitro observations, the combination of ERRα knockdown and etoposide treatment displayed synergistic effects in suppressing tumor growth, reducing tumor volume, and decreasing tumor weight in syngeneic 4T1 allograft models (Fig. 7K–M).

### ERRα correlates with type I IFN pathway suppression in breast cancer

**Fig. 8:**
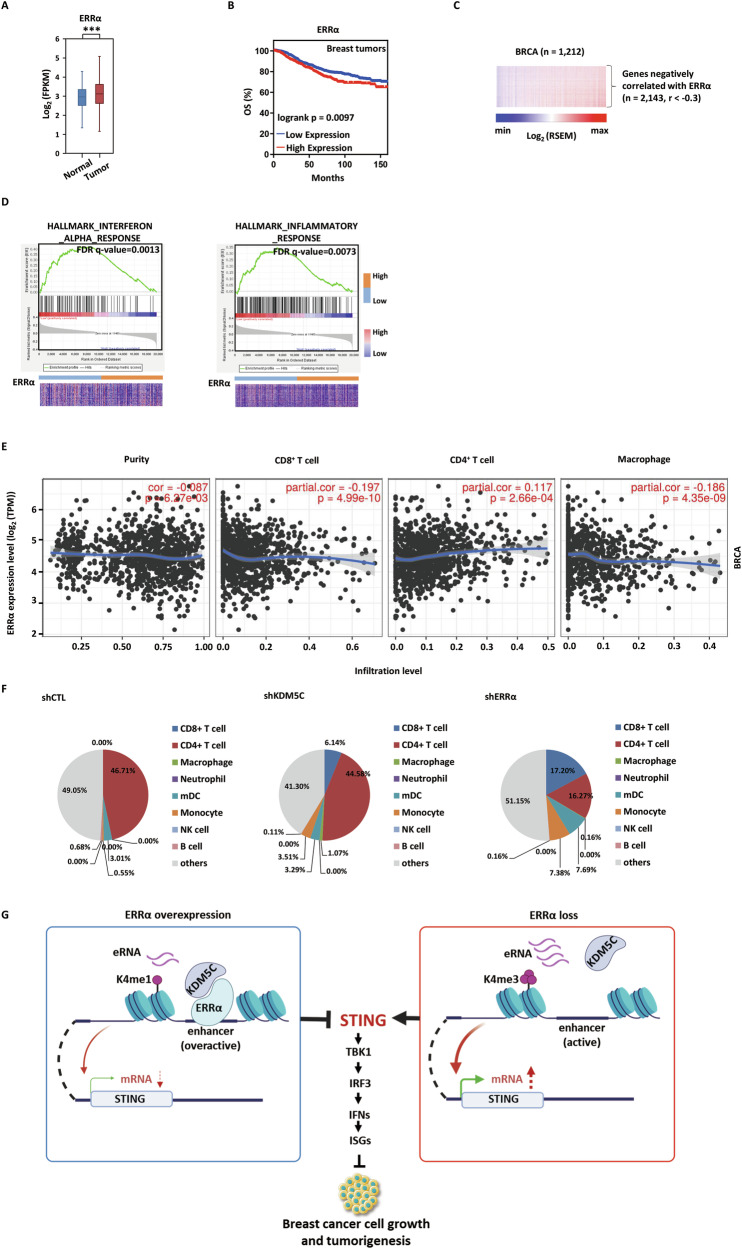
ERRα correlates with type I IFN pathway suppression in breast cancer. **A** Expression of ERRα in normal and breast cancer tissues (dataset as indicated). *P*-value was calculated by unpaired two-tailed Student’s *t-*test. **B** Overall survival (OS) of breast cancer patients was analyzed using the Kaplan–Meier Plotter database, stratified by ERRα expression (high, *n* = 654; low, *n* = 1225). **C** Genes that are negatively (*n* = 2143, r < −0.3) correlated with ERRα in BRCA (*n* = 1212) from TCGA are shown. RSEM: RNA-Seq by Expectation-Maximization. **D** GSEA was performed on gene expression profiles from TCGA-BRCA breast tumor samples with low (*n* = 100) or high (*n* = 100) ERRα expression (as indicated). Columns represent ERRα-low (blue bar) and ERRα-high (orange bar) tumor samples, and rows represent genes. **E** The correlation between ERRα expression and immune cell infiltration in breast tumors by TIMER analysis is shown. **F** The relative proportions of immune cell types in different experimental groups (shCTL, shKDM5C, and shERRα), as inferred from gene expression profiles based on RNA-seq data using the CIBERSORT deconvolution algorithm. **G** A proposed model depicts that in breast tumor cells, the highly expressed ERRα interacts with KDM5C to restrain STING enhancer activity, modulating type I IFN and ISG expression underlying antitumor immune responses and breast cancer progression.

The clinical relevance of ERRα-mediated repression of type I IFN responses was highlighted by its expression in multiple types of cancers, particularly in breast cancer. The expression of ERRα in clinical breast cancer samples was significantly higher than that in normal breast tissues (Fig. 8A). High expression of ERRα was significantly associated with poor prognosis in breast cancer patients (Fig. 8B). To elucidate the implications of ERRα-mediated transcriptional regulation in antitumor immunity, we conducted comprehensive transcriptomic profiling of breast cancer (BRCA) specimens from The Cancer Genome Atlas (TCGA) pan-cancer cohort. Through integrative bioinformatics analysis, we identified a significant inverse correlation between ERRα expression signatures and type I IFN response pathways (Fig. 8C, D). We found 2,143 genes negatively associated with ERRα using the Morpheus tool (Fig. 8C). GSEA revealed significant enrichment in IFN and inflammation signaling pathway (Fig. 8D). Using TIMER, we performed a correlation analysis between ERRα expression and CD8^+^ T cell infiltration, and found a significant negative correlation between ERRα expression and CD8^+^ T cell infiltration (Fig. 8E), which is in line with the immune cell composition estimated from RNA-seq data using the CIBERSORT algorithm (Fig. 8F).

## Discussion

ERRα, a nuclear receptor known to promote breast cancer progression through metabolic reprogramming [46–48], cell cycle regulation [49], and conferring resistance to cell death [50], is established in this study to form a functional complex with KDM5C. This complex localizes to the nucleus and represses STING enhancer activity, thereby dampening the STING-dependent type I IFN pathway and facilitating malignant proliferation (Fig. 8G). This KDM5C-ERRα-STING axis represents a previously unrecognized epigenetic-transcriptional nexus that links metabolic regulation (via ERRα) to innate immune signaling (via STING) within the tumor microenvironment, thereby impacting breast tumorigenesis through a non-canonical pathway. We note that the proliferative contribution of ERRα-KDM5C complex seems to exhibit context-dependence. The attenuated rescue phenotype observed in MDA-MB-231 cells compared to MCF7 cells (Fig. 6M–O) suggests the involvement of cell-line-specific regulatory mechanisms, likely attributable to their distinct molecular backgrounds. This implies that in MDA-MB-231 cells, dominant alternative survival pathways may supersede the STING-centric mechanism central to our model.

KDM5C is a critical regulator of enhancer activity, modulating H3K4 methylation dynamics. Our findings reveal that ERRα modulates the levels of H3K4me1/H3K4me3 at STING enhancers and their associated transcriptional activity through KDM5C. In breast cancer, KDM5C participates in the regulation of diverse immune response and migration-related pathways. Our data further demonstrate that ERRα is also involved in immune response modulation, suggesting that the ERRα-mediated KDM5C axis may contribute to immune evasion in breast cancer. Specifically, we observed that high ERRα expression restrains STING enhancer activity and transcription, suppresses type I IFN pathway, and correlates with reduced CD8^+^ T cell infiltration (Fig. 8E). Although this study centers on the function of KDM5C in modulating the type I IFN pathway in breast cancer, its paralogs, KDM5A [51] and KDM5B [52–54], are also significantly expressed in malignancy and contribute to tumor progression. Among them, KDM5B has been particularly well-characterized as a suppressor of the type I IFN pathway and antitumor immunity, with evidence spanning breast [55], prostate [56], and melanoma [57] cancers. Mechanistic insights reveal that KDM5B inhibition can remodel the immune microenvironment in pancreatic cancer through STING upregulation [58], raising the possibility of functional redundancy among KDM5 family members.

In summary, we identified a regulatory mechanism involving ERRα, which recruits KDM5C to dampen STING enhancer overactivation. This inhibition of the STING-mediated type I IFN pathway disrupts immune surveillance, thereby enabling cancer cells to evade immune detection and undergo malignant proliferation.

## Materials and methods

### Cell culture

MCF7, MDA-MB-231, HCC1937, 4T1, and HEK293T cells were obtained from the American Type Culture Collection (ATCC). MCF7, MDA-MB-231, and HEK293T cells were cultured in Dulbecco’s modified Eagle’s medium (DMEM) (Biological Industries, 01-052-1ACS) supplemented with 10% fetal bovine serum (FBS) (Biological Industries, 04-001-1ACS) and 1% penicillin/streptomycin (BasalMedia, S110JV). HCC1937 and 4T1 cells were cultured in RPMI 1640 medium (Biological Industries, 01-100-1ACS) supplemented with 10% FBS and 1% penicillin/streptomycin (BasalMedia, S110JV). All cells were maintained in a 5% CO_2_ humidified incubator at 37°C.

### Plasmids transfection, lentivirus packaging, and infection

Full-length or truncated constructs of KDM5C were as reported previously [35]. Short hairpin RNAs (shRNAs) targeting human or murine ERRα (shERRα-1, GACCTCTTTGACCGAGAGATT; shERRα-2, GAGAGGAGTATGTTCTACTAA; shERRα(m)-1, GCAGAGCAATAACACTATATT; shERRα(m)-2, GCAGGCAAAGTCCTGGCCCAT), human KDM5C (shKDM5C-1, AGTACCTGCGGTATCGGTATA; shKDM5C-2, GCCACACTTGAGGCCATAATC), and human STING (shSTING, GCATGGTCATATTACATCG) were cloned into pLKO.1-TCR cloning vector (Addgene, 10878). The constructs for wild-type KDM5C and its catalytically inactive mutant (H514A) have been described previously [35]. HEK293T cells were transfected with the mixture of the lentiviral and packaging vectors, psPAX2 (Addgene, 12260) and pMD2.G (Addgene, 12259), using polyethylenimine (PEI) (Polysciences, 24765-2) following the manufacturer’s instructions. Lentivirus-containing supernatants were harvested 60 h post-transfection. Viruses were collected and added in the presence of 10 μg/ml polybrene (Sigma, H9268). The medium was replaced 24 h later.

### RNA isolation and RT-qPCR

Total RNA was extracted from cells 72 h after infection with the indicated lentiviral vectors using the RNA-easy Isolation Reagent (Vazyme Biotech, R701-01) according to the manufacturer’s instructions. First-strand cDNA synthesis from total RNA was performed using the ABScript Neo RT Master Mix for qPCR with gDNA Remover Kit (ABclonal, RK20433), followed by quantitative polymerase chain reaction (qPCR) using BrightCycle Universal SYBR Green qPCR Mix with UDG (ABclonal, RK21219) on an AriaMx Real-Time PCR machine (Agilent Technologies). Sequence information for all primers used to check gene expression is presented in Supplementary Table S1.

### RNA sequencing (RNA-seq)

The RNA-easy Isolation Reagent (Vazyme Biotech, R701-01) was used for RNA isolation. DNase I was performed to ensure RNA quality. RNA library preparation was performed using the NEBNext^®^ UltraTM Directional RNA Library Prep Kit for Illumina (Illumina, E7420L). Paired-end sequencing was performed using an Illumina NovaSeq system. Sequencing reads were preprocessed using fastp for quality filtering and adapter removal, and subsequently aligned to the hg19 RefSeq database using Hisat2 (https://daehwankimlab.github.io/hisat2/). Cuffdiff was used to quantify the expression of RefSeq annotated genes with the option -M (reads aligned to repetitive regions were masked) and -u (multiple aligned reads were corrected using the “rescue method”). Coding genes with FPKM (fragments per kilobase per million mapped reads) >0.5 in at least one condition were included in our analysis. Up- or down-regulated genes were determined by the fold change (FC) of the gene’s FPKM (FC > 1.5). The FPKM of gene was calculated as mapped reads on exons divided by exonic length and the total number of mapped reads. Box plots were generated using Microsoft Excel, and statistical significance was determined using Student’s *t*-test. Heatmaps were visualized with the Java TreeView software. Functional enrichment analysis was conducted using Metascape (https://metascape.org) for Reactome pathway enrichment, integrating hypergeometric testing and functional annotation. The immune cell composition of shCTL, shKDM5C, and shERRα breast cancer cells was inferred from RNA-seq expression profiles using the TIMER 2.0 platform (http://timer.cistrome.org/). The relative fractions of each immune cell type were summarized from TIMER output and visualized as pie charts in Microsoft Excel, displaying the comparative distributions of CD8^+^ T cells, CD4^+^ T cells, macrophages, neutrophils, monocytes, myeloid dendritic cells (mDCs), natural killer (NK) cells, B cells, and others. ssGSEA was performed using the ssGSEAProjection module on the GenePattern public server (https://cloud.genepattern.org/). Protein-coding genes with an RPKM value greater than 0.5 in at least one of the three RNA-seq samples (shCTL, shKDM5C, and shERRα) were included in the analysis. Gene sets were derived from the Molecular Signatures Database (MSigDB) Hallmark collections. For each sample, ssGSEA generated an enrichment score representing the relative activation level of each pathway independent of biological replicates. The average Δscore between shCTL and shKDM5C or shERRα groups was calculated for each pathway to rank the enrichment changes. The top five pathways with the largest mean Δscores were selected for visualization. Heatmaps were generated using Morpheus (https://software.broadinstitute.org/morpheus/). Before visualization, enrichment scores were normalized by subtracting the row median and dividing by the row median absolute deviation, as recommended by the Morpheus software.

### Chromatin immunoprecipitation sequencing (ChIP-seq) and ChIP-qPCR

Cells were fixed with 1% formaldehyde (Sigma, 689316) for 10 min at room temperature (RT), washed twice with PBS. Fixation was quenched by adding glycine (0.125 M) and incubated for 5 min at RT, followed by two washes with cold PBS. Chromatin DNA was sheared to 200–500 bp average in size through sonication in ChIP lysis buffer (1% SDS, 10 mM EDTA, 50 mM Tris-HCl pH 7.8, and 1×protease inhibitor cocktail (Sigma, P2714)) as described previously [35]. The resultant was diluted in dilution buffer (1% Triton X-100, 2 mM EDTA, 150 mM NaCl, 20 mM Tris-HCl, pH 7.8, and 1×protease inhibitor cocktail) and immunoprecipitated with specific antibodies overnight at 4 °C, followed by incubation with Protein A/G magnetic beads (ABclonal, RM02915) for an additional 2 h at 4 °C with rotation. The bound fractions were sequentially washed for 15 min at 4 °C with TSEI (0.1% SDS, 1% Triton X-100, 2 mM EDTA, 150 mM NaCl, 20 mM Tris-HCl pH 7.8, and 1×protease inhibitor cocktail), TSEII (0.1% SDS, 1% Triton X-100, 2 mM EDTA, 400 mM NaCl, 20 mM Tris-HCl pH 7.8, and 1×protease inhibitor cocktail), and TE buffer (20 mM Tris-HCl pH 7.4, and 1 mM EDTA). Formaldehyde crosslinked chromatin was treated at 65 °C overnight with interval shaking. Immunoprecipitated DNA was purified by using AFTSpin Multifunction DNA Purification Kit (ABclonal, RK30100) and subjected to high-throughput sequencing or qPCR. The Relative enrichment fold the ChIP/CUT&Tag-qPCR data was determined using the percent input method. Specifically, the signal from the immunoprecipitated sample was first normalized to its corresponding input DNA. This normalized value was then compared to the mean normalized value from a control/reference sample to calculate the final fold enrichment.

### Global run-on sequencing (GRO-seq)

GRO-seq was performed in MCF7 cells according to a previously described protocol [59] with the following procedures. Cells were washed three times with ice-cold PBS and incubated in swelling buffer (10 mM Tris-Cl, pH 7.5, 2 mM MgCl₂, 3 mM CaCl₂) for 5 min on ice before harvesting. Nuclei were released by resuspending the cell pellet in lysis buffer (swelling buffer supplemented with 0.5% IGEPAL and 10% glycerol). The isolated nuclei were washed once with 10 mL of lysis buffer and resuspended in 100 μL of freezing buffer (50 mM Tris-Cl, pH 8.3, 40% glycerol, 5 mM MgCl₂, 0.1 mM EDTA). For the nuclear run-on assay, the nuclei were mixed with an equal volume of reaction buffer (10 mM Tris-Cl, pH 8.0, 5 mM MgCl₂, 1 mM DTT, 300 mM KCl, 20 U of SUPERase In, 1% sarkosyl, 500 µM ATP, GTP, and Br-UTP, and 2 µM CTP) and incubated at 30 °C for 5 min. Newly transcribed BrU-labeled RNA was extracted using TRIzol LS reagent (Invitrogen) according to the manufacturer’s instructions. The nuclear run-on RNA (NRO-RNA) was hydrolyzed on ice for 40 min, treated with DNase I, and dephosphorylated with Antarctic phosphatase. BrU-labeled nascent RNA was immunoprecipitated with anti-BrdU agarose beads (Santa Cruz Biotechnology) in binding buffer (0.5×SSPE, 1 mM EDTA, 0.05% Tween-20) for 1 h at 4 °C with rotation. The immunoprecipitated RNA was subsequently treated in a 50 μL end-repair reaction containing 43 μL DEPC-treated H₂O, 5.2 μL T4 PNK buffer, 1 μL SUPERase In, and 1 μL T4 polynucleotide kinase (New England BioLabs) for 1 h at 37 °C. RNA was then recovered by acidic phenol-chloroform (Ambion) extraction and ethanol precipitation. A poly(A) tail was added to the RNA using poly(A) polymerase (New England BioLabs) for 30 min at 37 °C. Reverse transcription was performed using SuperScript III (Invitrogen) with the primer oNTI223 (5′-pGATCGTCGGACTGTAGAACTCT; CAAGCAGAAGACGGCATACGATTTTTTTTTTTTTTTTTTTTVN-3′), where “p” indicates 5′ phosphorylation, “;” denotes an abasic dSpacer furan, and “VN” represents degenerate nucleotides. cDNA products were separated on a 10% polyacrylamide TBE-urea gel, and fragments ranging from 100 to 500 bp were excised and purified. The first-strand cDNA was circularized using Circligase (Epicentre) and subsequently relinearized with Ape1 (New England BioLabs). The relinearized single-stranded cDNA was size-selected (170–400 bp) on a 10% polyacrylamide TBE gel, extracted, and amplified by PCR with Phusion High-Fidelity DNA Polymerase (NEB) using primers oNTI200 (5′-CAAGCAGAAGACGGCATA-3′) and oNTI201 (5′-AATGATACGGCGACCACCGACAGGTTCAGAGTTCTACAGTCCGACG-3′). Final libraries were sequenced on an Illumina HiSeq 2000 platform with the small RNA sequencing primer 5′-CGACAGGTTCAGAGTTCTACAGTCCGACGATC-3′.

### Computational analysis of ChIP-seq and GRO-seq data

The ChIP-seq data for KDM5C and ERRα were reanalyzed from publicly available datasets, including our previous study [35] and the ENCODE dataset (GEO accession GSM2424192, Snyder Lab, Stanford University). ChIP-seq and GRO-seq reads were aligned to the human reference genome (hg19) using Bowtie2 (http://bowtie-bio.sourceforge.net/bowtie2/index.shtml) with default parameters. Both uniquely aligned reads and reads mapping to repetitive regions were retained for downstream analysis. In cases where a read aligned to multiple genomic locations, only the alignment with the highest mapping score was retained. To mitigate the impact of clonal amplification, a maximum of one tag per unique genomic position was allowed. Peak identification for ChIP-seq data was performed using HOMER (http://homer.ucsd.edu/homer/), where ChIP-seq reads were extended by 200 bp to account for the average fragment size. Peaks were defined as regions with a tag enrichment significantly above background, utilizing an input control to ensure accurate peak identification. A minimum four-fold tag enrichment relative to the input control was required for each putative peak. Additionally, a Poisson p-value threshold of 0.0001 was applied to assess the statistical significance of tag count differences. Co-occupancy was defined as loci where KDM5C and ERRα peak center overlapped within ≤600 bp, both satisfying these significance thresholds. Genomic distribution analysis was conducted with HOMER, using modified parameters to define promoter peaks as those with centers located within 200 bp downstream to 5000 bp upstream of a transcription start site (TSS). Enhancers were defined as peaks located outside of promoter regions. Heatmaps were generated and visualized using deepTools (https://deeptools.readthedocs.io/en/latest/) or Morpheus. Tag density histograms were generated with 50 bp bins using deepTools or HOMER, while tag density scatter plots and box plots were created with HOMER.

### Immunoprecipitation and immunoblotting

For immunoblotting, cells were collected and lysed in RIPA buffer (50 mM Tris-HCl, pH 7.4, 150 mM NaCl, 0.1% SDS, 0.5% sodium deoxycholate (Sangon, A100613), and 1% Noidet-P-40) with protease inhibitor cocktail (Sigma, P2714). Cell lysates were incubated on ice for 10 min, and briefly sonicated to shear chromatin, then cleared by high-speed centrifugation (20,000 × *g*, 30 min) at 4 °C. The supernatants were used for analysis. The protein concentration was quantified by Bradford method. The lysates mixed with SDS loading buffer were heated at 95 °C for 10 min. 30–60 μg of total proteins were subjected to SDS-PAGE analysis. The proteins were transferred onto PVDF membrane (Vazyme Biotech, E801-01) and blocked with 5% skimmed milk for 1 h at RT. Then, the membrane was incubated overnight with primary antibodies dissolved in 5% skimmed milk-TBST (50 mM Tris-HCl, pH 7.6, 150 mM NaCl, 0.1% Tween-20). After overnight incubation with primary antibodies, the membrane was washed three times with 1×TBST buffer (10 min per wash). A secondary antibody was prepared in 5% skim milk at a 1:5000 dilution and incubated with the membrane at room temperature for 1 h. Following this, the secondary antibody solution was discarded, and the membrane was washed three additional 10-min washes with 1×TBST buffer at RT. Immunopositive bands were detected using an ECL kit (Advansta, K-12045-D10) and captured using ChemiDoc XRS chemiluminescence imaging system (Bio-Rad).

For endogenous KDM5C or ERRα immunoprecipitation, MCF7 cells were snap-frozen in liquid nitrogen and lysed in lysis buffer containing 50 mM Tris-HCl (pH 7.5), 250 mM NaCl, 5 mM EDTA, 0.5% NP-40, and complete protease inhibitor cocktail (Sigma). For each immunoprecipitation, 200 μg of clarified supernatant was incubated with 2 μg of the indicated antibody at 4 °C overnight with gentle rotation. Protein A/G agarose beads (Beijing LABLEAD lnc.) were then added, and the mixture was incubated for an additional 2 h at 4 °C. The beads were subsequently washed five times with lysis buffer and then resuspended in SDS sample buffer (1% SDS, 5% glycerol, 50 mM DTT, 30 mM Tris-HCl, pH 6.8, 0.25% bromophenol blue) and boiled for 5 min at 95–100 °C.

### Immunoprecipitation–LC-MS/MS analysis

Protein immunoprecipitation was performed following the protocol described previously [35]. MCF7 cells were infected with lentiviral vectors expressing pCDH-CMV-3×Flag-KDM5C-3×HA-EF1-Puro for 48 h. Subsequently, nuclear extracts were prepared using Buffer A (10 mM HEPES-KOH, pH 7.8, 1.5 mM MgCl₂, 10 mM KCl, 1 mM DTT, and 1×complete protease inhibitor cocktail). The nuclear extracts were lysed in Buffer C (20 mM HEPES-KOH pH 7.8, 25% glycerol, 420 mM NaCl, 1.5 mM MgCl₂, 0.2 mM EDTA pH 8.0, 1 mM DTT, and 1×complete protease inhibitor cocktail) and subjected to affinity purification using anti-HA agarose (Beijing LABLEAD lnc.). The beads were washed extensively three times with Buffer D (0.15) (150 mM KCl, 20 mM HEPES-KOH pH 7.8, 15% glycerol, 0.2 mM EDTA pH 8.0, 0.2% NP-40, 1 mM DTT, and 1×complete protease inhibitor cocktail), followed by two washes with Buffer D (0.1) (100 mM KCl, 20 mM HEPES-KOH pH 7.8, 15% glycerol, 0.2 mM EDTA pH 8.0, 0.2% NP-40, 1 mM DTT, and 1×complete protease inhibitor cocktail). Proteins were eluted using HA peptides (Sigma). The eluates were digested in solution and analyzed by LC-MS/MS. Briefly, immunoprecipitated eluates were reduced with 20 mM DTT (Sigma) at 95 °C for 5 min and alkylated with 50 mM iodoacetamide (Sigma) in the dark at room temperature for 30 min. Samples were then transferred to a 10 kDa centrifugal spin filter (Millipore) and washed three times with 200 μL of 8 M urea and twice with 200 μL of 50 mM ammonium bicarbonate by centrifugation at 14,000 × *g*. Tryptic digestion was carried out by adding trypsin (Promega) at a 1:50 (enzyme-to-substrate) ratio in 200 μL of 50 mM ammonium bicarbonate and incubating at 37 °C for 16 h. Peptides were collected by centrifugation at 14,000 × *g*, and the filter was washed twice with 100 μL of 50 mM ammonium bicarbonate to improve peptide recovery. Peptides were desalted using StageTips. LC-MS/MS analysis was performed on a nanoscale UHPLC system (EASY-nLC 1000, Proxeon Biosystems) coupled to an Orbitrap Q-Exactive mass spectrometer (Thermo Fisher Scientific) equipped with a nanoelectrospray ion source. Peptides were dissolved in 0.1% formic acid (FA) with 2% acetonitrile (ACN) and separated on a reverse-phase analytical column (75 μm × 15 cm) packed with 2 μm C18 beads (Thermo Fisher Scientific) using a 4-h gradient from 5% to 35% ACN in 0.1% FA at a flow rate of 300 nL/min. The spray voltage was set to 2.5 kV, and the ion transfer capillary temperature was maintained at 275 °C. Full MS scans were acquired at a resolution of 70,000 over m/z 350–1800, with an AGC target of 1e6 and a maximum injection time of 100 ms. The top twenty most intense ions were selected for fragmentation via HCD with a normalized collision energy of 28%, and MS/MS spectra were acquired at a resolution of 17,500 (AGC target 1e5, maximum injection time 100 ms). Dynamic exclusion was set to 30 s. Raw data were processed using Proteome Discoverer (version 2.1), and MS/MS spectra were searched against the Swiss-Prot human database. Search parameters included a precursor mass tolerance of 10 ppm, a fragment mass tolerance of 0.02 Da, variable modifications (oxidation of methionine, methylation and dimethylation of arginine/lysine, and N-terminal acetylation), fixed carbamidomethylation of cysteine, and allowance for up to three missed tryptic cleavages. Peptides were required to have at least six amino acids. Peptide and protein identifications were filtered to achieve a false discovery rate (FDR) of less than 1%, with at least one unique peptide required for high-confidence protein identification.

### Antibodies

Antibodies used are listed as following: Rabbit anti-KDM5C (Bethyl Laboratory, A301-034A, 1:100 for IP), Rabbit anti-ERRα (ABclonal, A14184, 1:100 for IP), Rabbit anti-ERRα (Cell Signaling Technology, 13826, 1:1000), Rabbit anti-KDM5C (Cell Signaling Technology, 5361, 1:1000), Mouse anti-β-ACTIN (ABclonal, AC004, 1:5000), Rabbit anti-STING (ABclonal, A3575, 1:1000), Rabbit anti-Phospho-TBK1 (Cell Signaling Technology, 5483, 1:1000), Rabbit anti-TBK1 (Cell Signaling Technology, 3504, 1:1000), Rabbit anti-Phospho-IRF3 (Cell Signaling Technology, 4947, 1:1000), Rabbit anti-Phospho-IRF3 (Cell Signaling Technology, 4302, 1:1000), Mouse anti-HA (ABclonal, AE008, 1:1000), Rabbit anti-TriMethyl-H3K4 (ABclonal, A22146, 1:100 for ChIP-seq), Rabbit anti-TriMethyl-H3K4 (ABclonal, A2357, 1:100 for ChIP and CUT&Tag), Rabbit anti-DiMethyl-H3K4 (ABclonal, A2356, for CUT&Tag), Rabbit anti-MonoMethyl-H3K4 (ABclonal, A2355, for ChIP), HRP-conjugated Goat anti-Mouse IgG (ABclonal, AS003, 1:10,000), HRP-conjugated Goat anti-Rabbit IgG (ABclonal, AS014, 1:80,000).

### Cleavage under targets and tagmentation (CUT&Tag)

CUT&Tag was performed with Hyperactive Universal CUT&Tag Assay Kit for Illumina Pro (Vazyme Biotech, TD904) according to the manufacturer’s instructions. In detail, 1 × 10⁵ cells were resuspended in binding buffer and incubated with 10 μL Concanavalin A (ConA) magnetic beads for 10 min at room temperature to immobilize cells. After magnetic separation and supernatant removal, cells were incubated with primary antibody (anti-H3K4me3, ABclonal A2357, 1 μg in 50 μL antibody buffer) overnight at 4 °C. Following two washes with dig-wash buffer (0.05% digitonin), samples were treated with secondary antibody (0.5 μL in 50 μL dig-wash buffer) for 1 h at room temperature. Unbound antibodies were removed by two additional dig-wash buffer washes. Tagmentation was initiated by adding pG-Tn5 transposase (2 μL preloaded with Illumina adapters) in 98 μL dig-300 buffer (20 mM HEPES pH 7.5, 0.5 mM spermidine, 0.05% digitonin, 300 mM NaCl) and incubating for 1 h at room temperature. Excess transposase was removed by two dig-300 buffer washes. To activate DNA fragmentation, 10 μL of 5×TTBL buffer (containing 10 mM Mg²⁺) was added to 40 μL dig-300 buffer, followed by incubation at 37 °C for 1 h. DNA purification was performed by adding 100 μL Buffer L/B, 5 μL Proteinase K, and 20 μL DNA extraction beads, with incubation at 55 °C for 10 min. DNA was magnetically purified and eluted in nuclease-free water. Library was assessed by qPCR amplification of target loci prior.

### Cell proliferation and colony formation

Cell proliferation assay was performed using CellTiter 96 AQueous one solution cell proliferation assay kit (Promega, G3582) following the manufacturer’s protocol. Briefly, 20 uL of CellTiter 96 AQueous one solution reagent was added to 100 uL culture medium and incubated for 1 h at 37 °C in a humidified at 5% CO_2_ atmosphere. The reaction was measured at wavelength 492 nm on Spark spectrophotometer (Tecan). For colony formation assay, cells were seeded in 6-well plates followed by lentivirus infection on the next day. Medium was replaced every 2 days. The relative cell number was determined by crystal violet staining. Briefly, cells were fixed with methanol/acetic acid solution (3:1) for 10 min and stained with 0.5% crystal violet (Sango Biotech, A100528-0025) for 30 min. For quantification, the crystal violet dye was released into 10% acetic acid and measured at wavelength 590 nm (OD590).

### Animal studies

Female BALB/c mice (age 4–6 weeks) were purchased from Beijing Vital River Laboratory Animal Technology, China, and maintained under standard laboratory conditions with 12 h light/12 h dark cycles and free access to standard rodent chow and water, in accordance with institutional guidelines. All of the animal experiments were approved by Animal Ethics Committee of Gannan Medical University (approval number: LLSC-2023-285).

### Mouse tumor models

For the 4T1 tumor allograft experiments, female BALB/c mice (4–6 weeks old) were subcutaneously implanted with 5 × 10⁵ 4T1 cells expressing shCTL or shERRα(m)-1/2 suspended in PBS. Tumor growth was monitored every 2 days using calipers once tumors became palpable, with both length (D) and width (d) recorded. Tumor volume was calculated using the ellipsoid formula: *V* = 1/2 × *D* × *d*^2^. Mice were euthanized when tumors reached 1500 mm³ in volume or upon the occurrence of tumor ulceration/bleeding. Excised tumors were then photographed and weighed. For etoposide treatment, mice were intraperitoneally administered etoposide (MedChemExpress, 8 mg/kg) every 2 days for a total of 6 doses. The size of tumors did not exceed the maximum limit set (1 cm in diameter) by the Animal Ethics Committee of Gannan Medical University.

### Integrated analysis of ERRα-associated genes, pathways, and immune infiltration in TCGA BRCA

Transcriptomic and clinical data for BRCA were obtained from TCGA cohort (TCGA-BRCA) (https://portal.gdc.cancer.gov/). RNA-seq FPKM data from tumor and adjacent normal tissues were analyzed using Microsoft Excel, and expression differences of ERRα (ESRRA) between tumor and normal samples were visualized as box plots. Survival analysis based on ERRα expression was performed using the Kaplan–Meier plotter database (https://kmplot.com/analysis/). This resource integrates gene expression and survival data from multiple public cohorts. Survival curves were exported and plotted in GraphPad Prism. Genes inversely associated with *ERRα* (*ESRRA*) expression were identified using Spearman correlation analysis (*ρ* < −0.3, adjusted *P* < 0.05) via the Morpheus tool (https://software.broadinstitute.org/morpheus/). A total of 2143 genes met significance thresholds and were selected for downstream analysis. GSEA analyses were conducted using GSEA software (Broad Institute) based on normalized FPKM expression values from TCGA-BRCA. The Hallmark gene sets from the MSigDB were used as reference, and enrichment scores were computed under default permutation settings (*n* = 1000). For group comparison, 200 breast tumor samples from the TCGA-BRCA cohort with ERRα (FPKM) expression values between 13.595 and 43.054 were selected. Within this range, samples were divided into high (*n* = 100) and low (*n* = 100) expression groups based on ranked FPKM values for subsequent GSEA analysis. Correlations between ERRα (transcripts per million, TPM) expression and the infiltration levels of CD8^+^ T cells, CD4^+^ T cells, and macrophages were assessed using TIMER 2.0, with tumor purity adjustment applied through partial correlation analysis.

### Quantification and statistical analysis

No statistical methods were used to predetermine sample size. Statistical analysis was performed with Microsoft Excel or Prism software (GraphPad Software). Data are presented as the means ± SEM (standard error of the mean). A two-tailed Student’s *t*-test was used to compare differences between experimental groups and their unpaired controls. Differences in compared groups were considered statistically significantly different with *P* values: ns: *P* ≥ 0.05; *P < 0.05; **P < 0.01; ***P < 0.001.

### Ethics approval and consent to participate

All animal experiments were performed in accordance with the relevant guidelines and regulations. All animal experimental procedures were reviewed and approved by the Ethics Committee of Gannan Medical University (approval number: LLSC-2023-285).

## Supplementary information

Original Western blots

Supplementary Figure Legends and Figures

Table S1. Primer sequences used for qPCR detection of mRNA and chromatin targets.

## Data Availability

The raw data of RNA-seq, GRO-seq, and ChIP-seq have been deposited in the Gene Expression Omnibus database under accession GSE300441. KDM5C ChIP-seq were from GSE141988; ERRα ChIP-seq were from GSE92187; the comparable input control ChIP-seq for the above public datasets were obtained from GSE62222 and used for peak identification and co-occupancy analysis. H3K27Ac ChIP-seq were from GSE62229; H3K9me3 and H3K27me3 ChIP-seq were from GSE23701. Further information and requests for resources and reagents should be directed to and will be fulfilled by the lead contact, W-JZ.

## Materials availability

All plasmids, reagents, and cell lines generated in this study are available from the lead contact.
