# Supplementary material for: ERRα-KDM5C restrains STING enhancer activity to modulate type I interferon signaling in breast cancer progression
Source: Cell Death Dis. 2026 Feb 18;17(1):228. doi: 10.1038/s41419-026-08499-2 (PMC12920621; doi:10.1038/s41419-026-08499-2)

Figure 1B

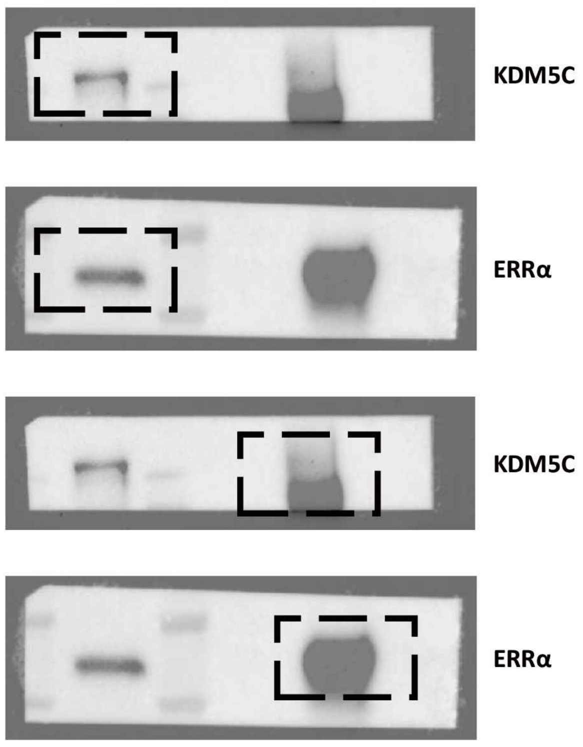

Figure 1C

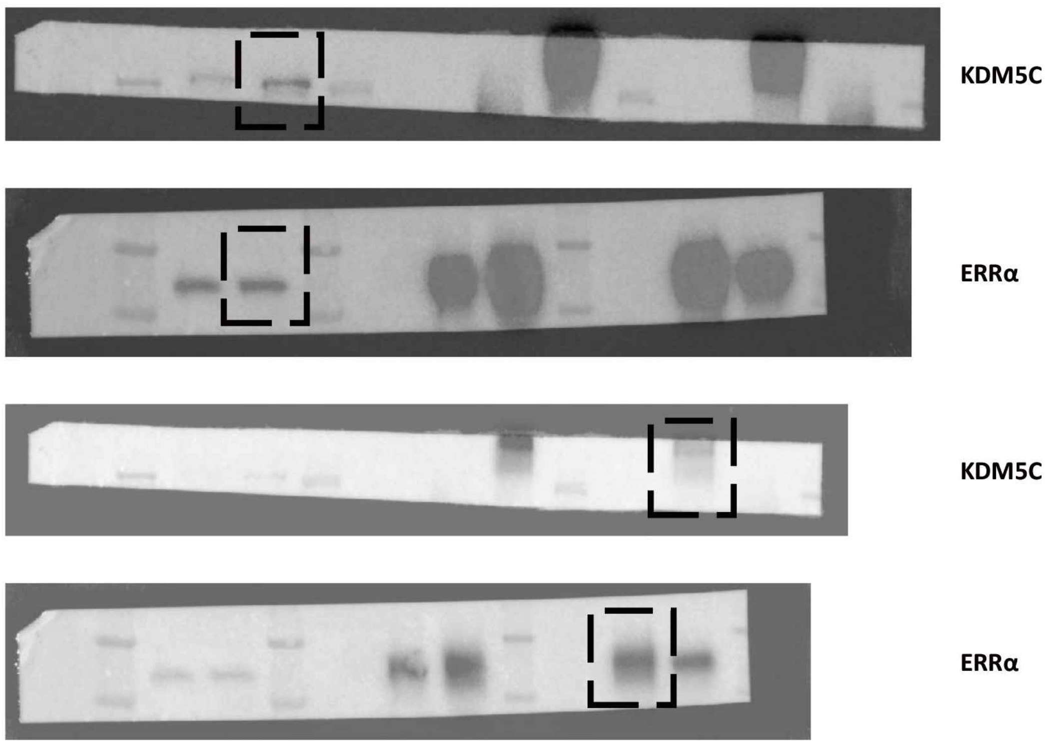

Figure 3D

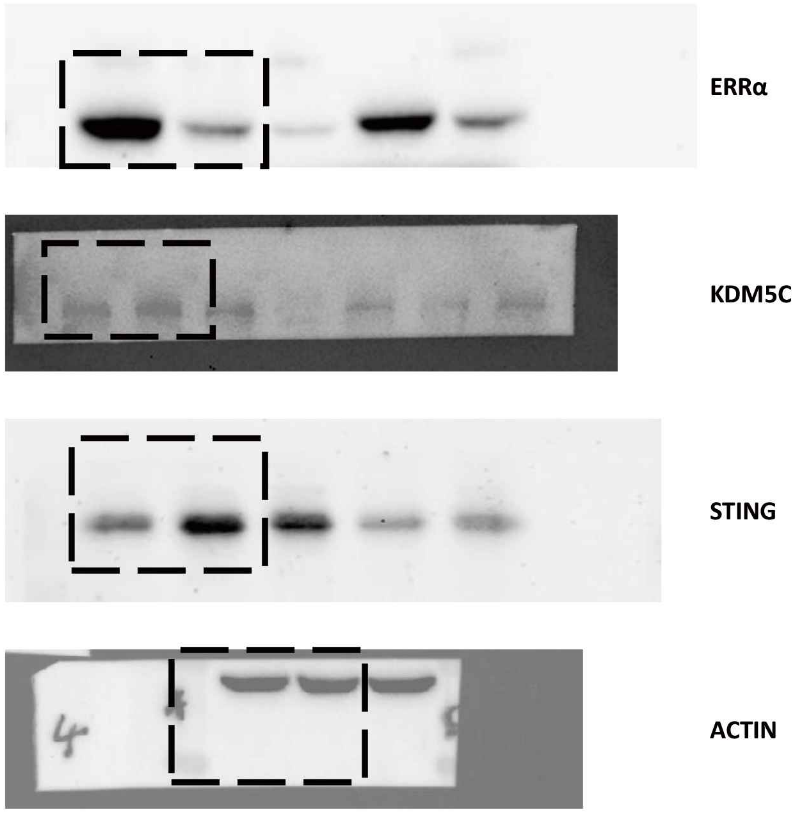

Figure 3E

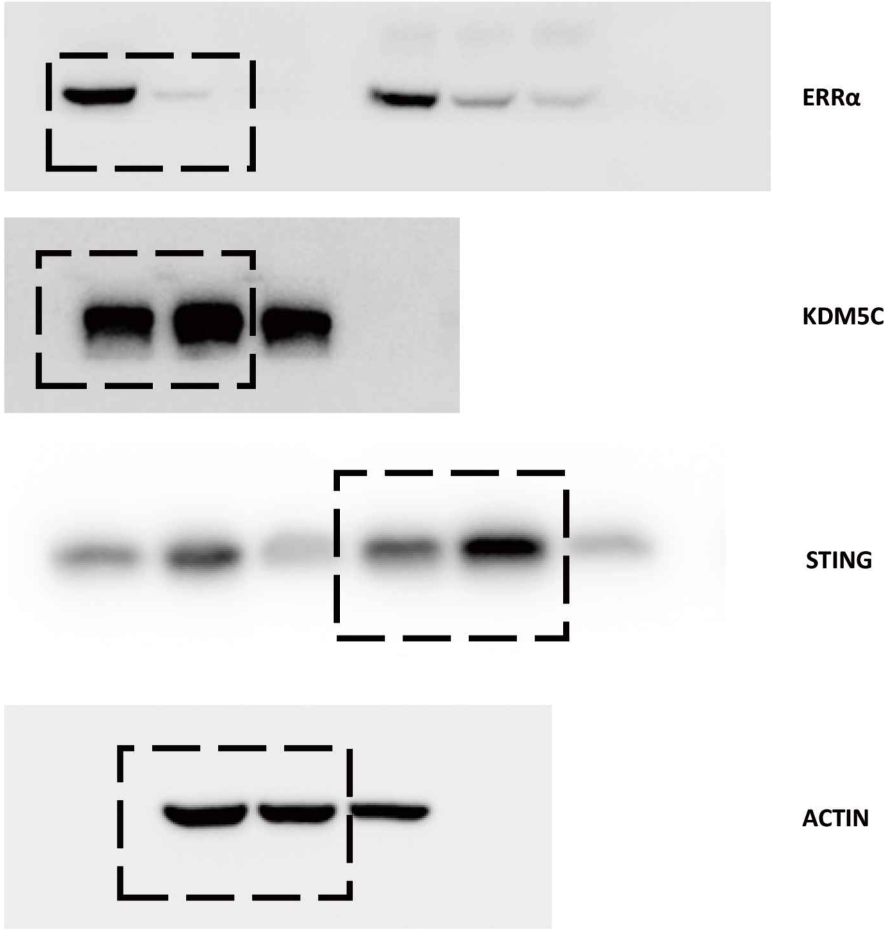

Figure 3F

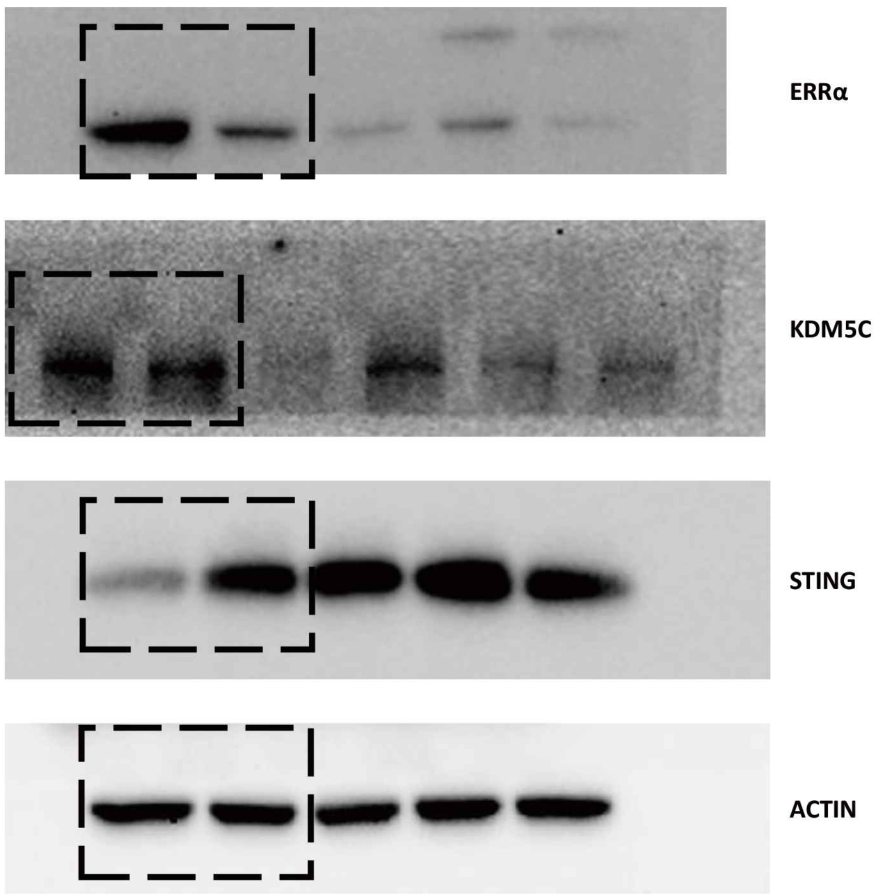

Figure 3H

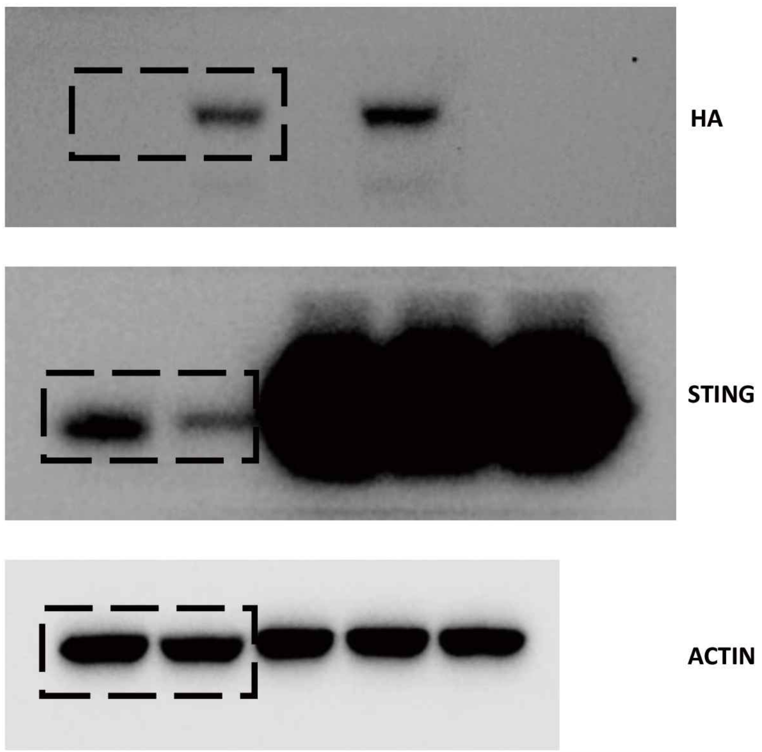

Figure 3I

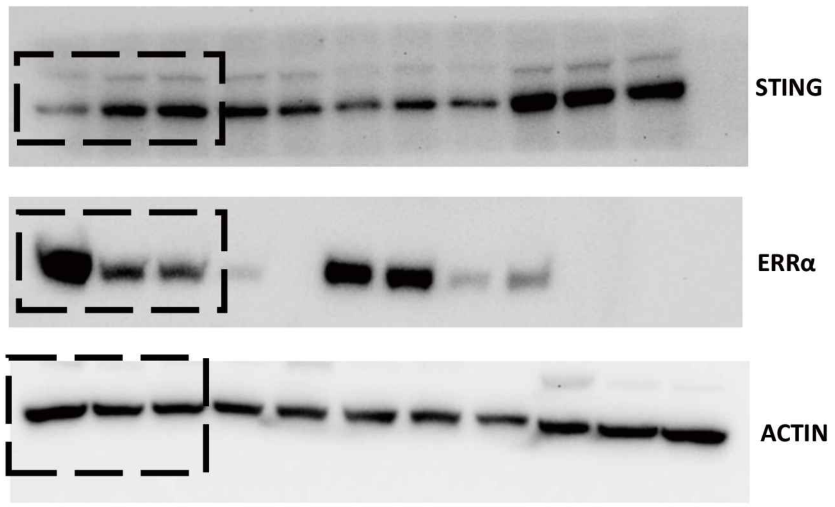

Figure 5B

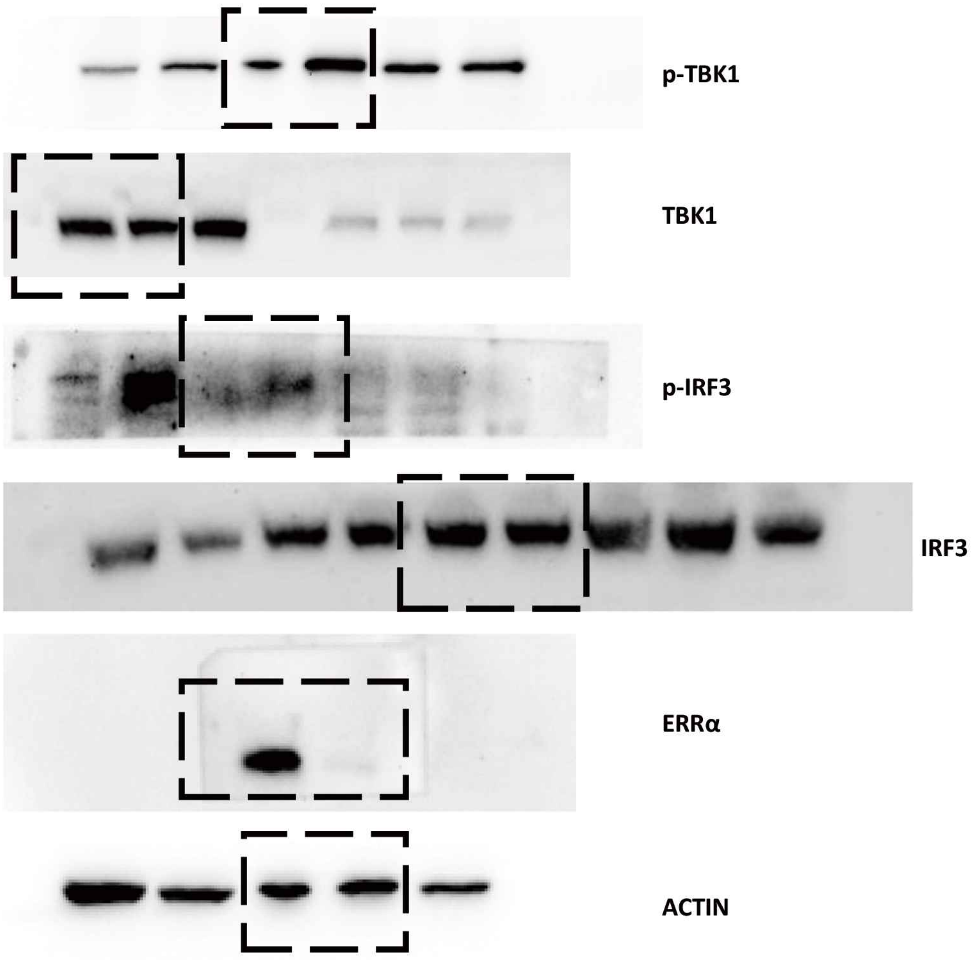

Figure 5D

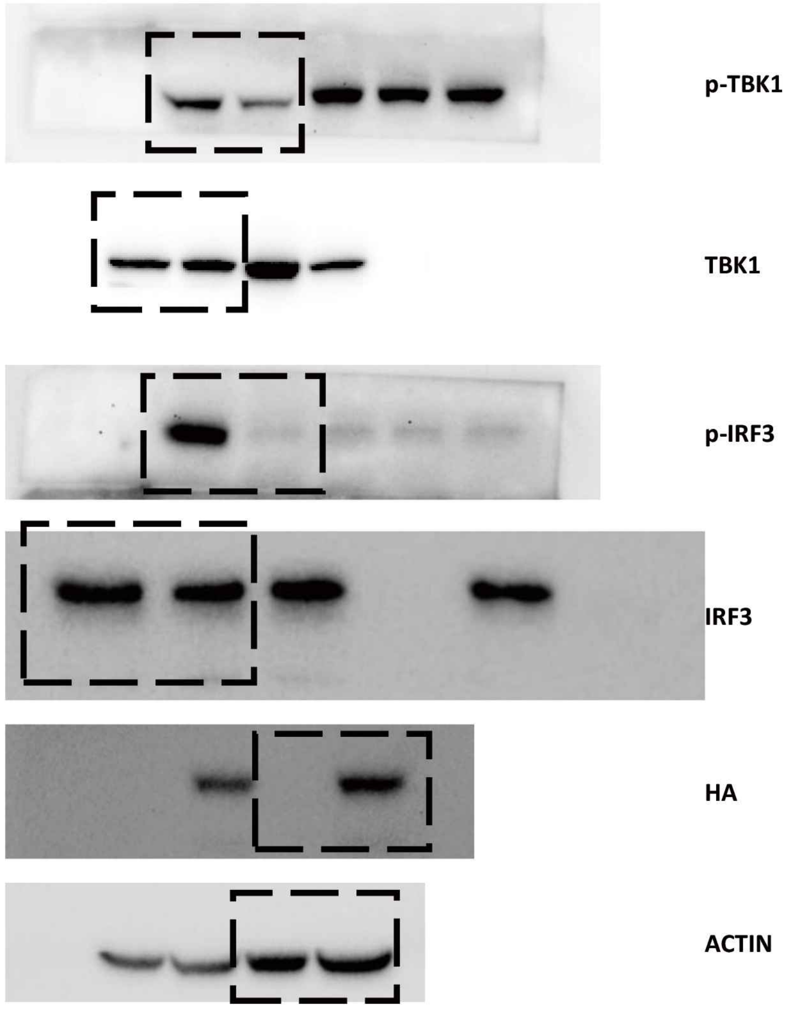

Figure 5A

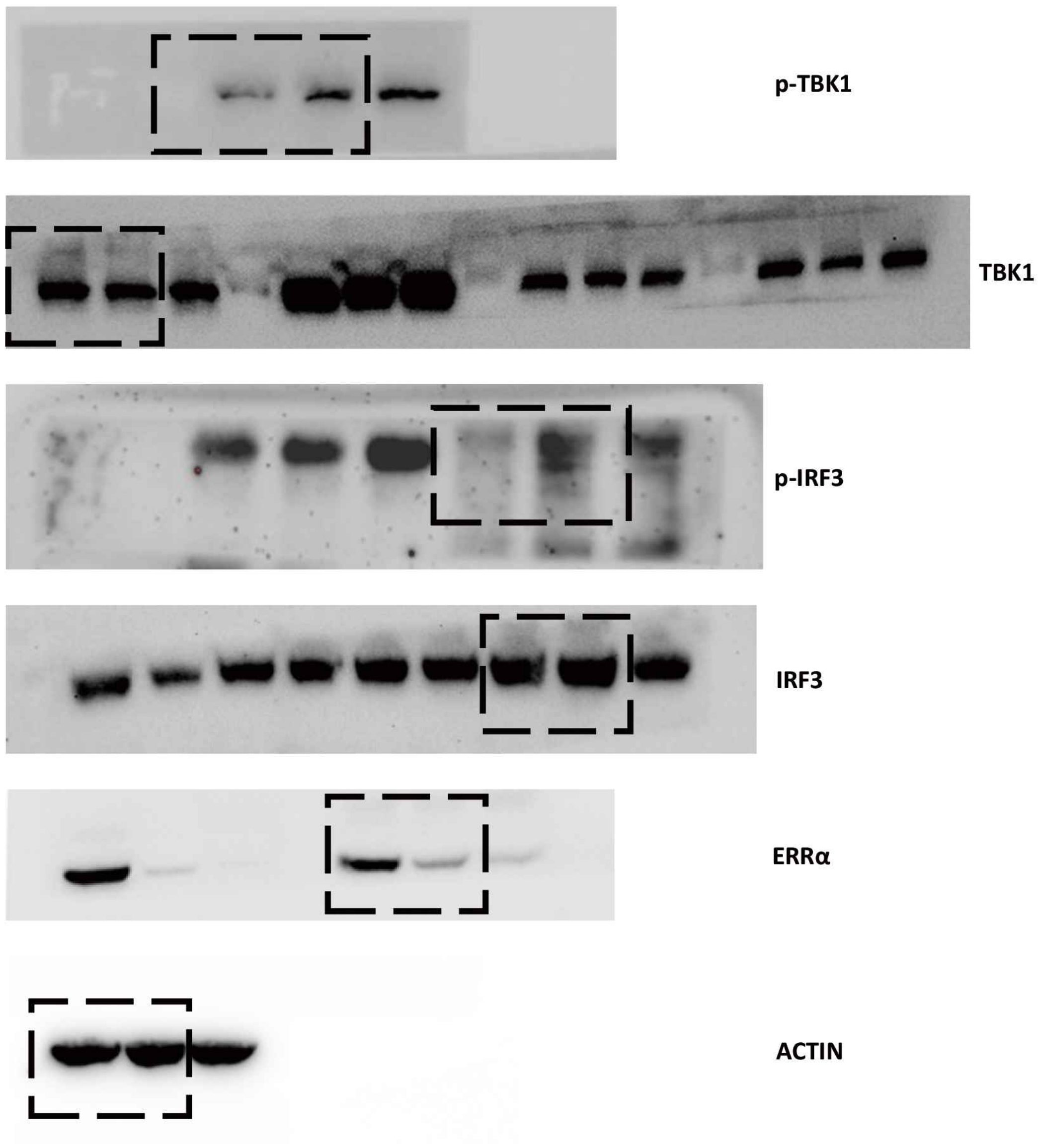

Figure 5C

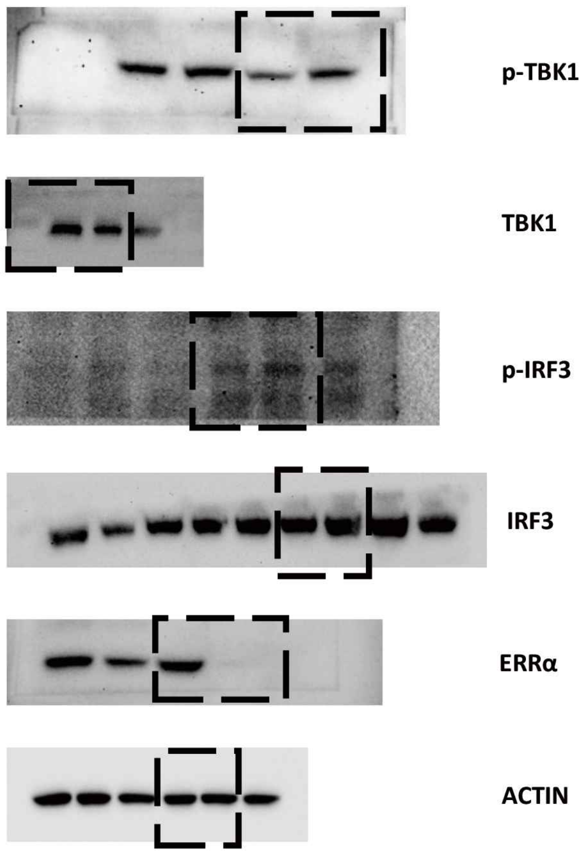

Figure 5E

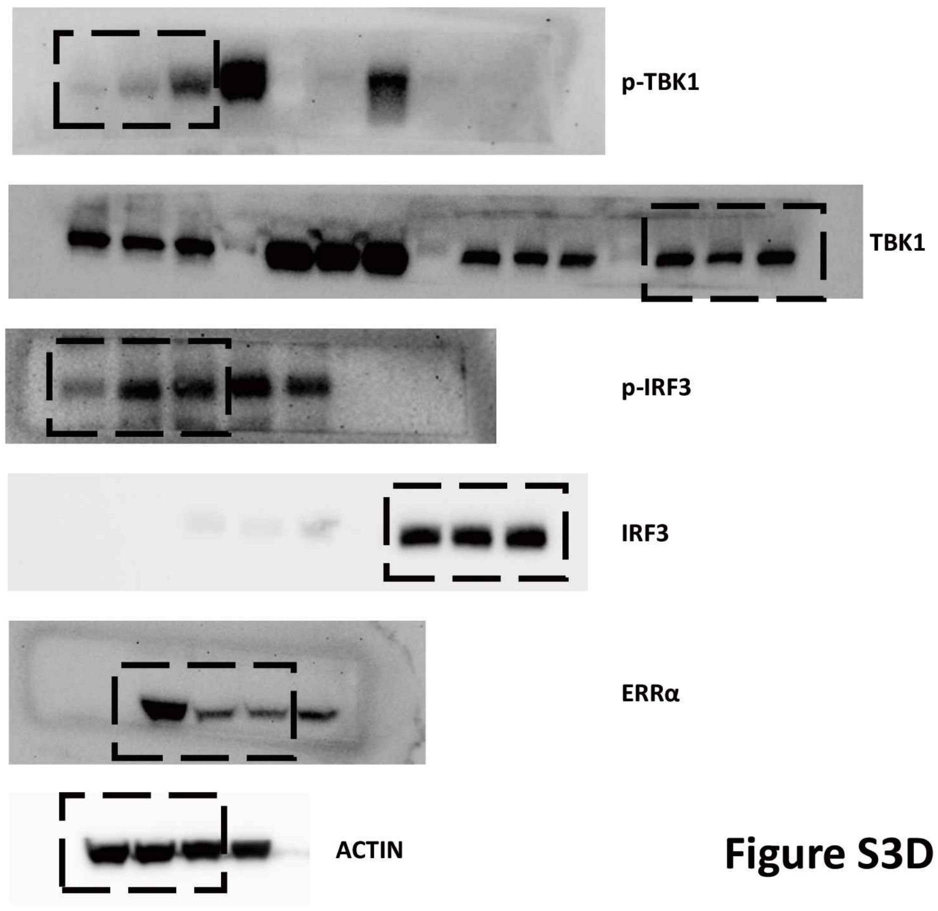

Figure 7G

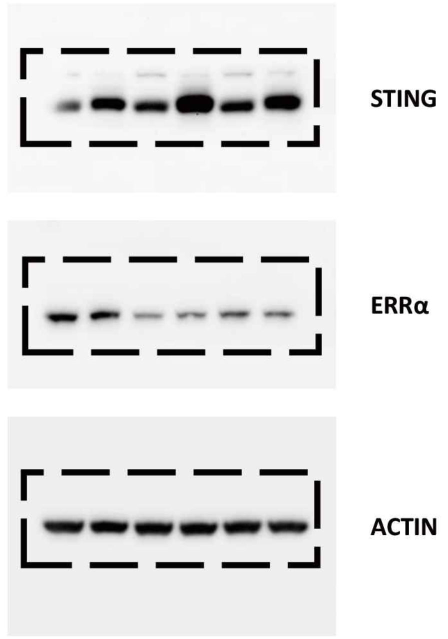

Figure S1A

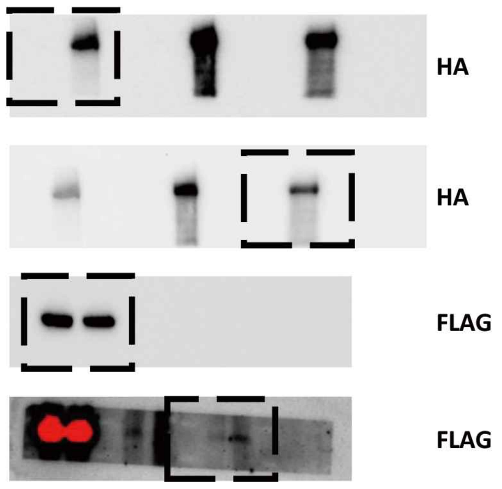

Figure S3D

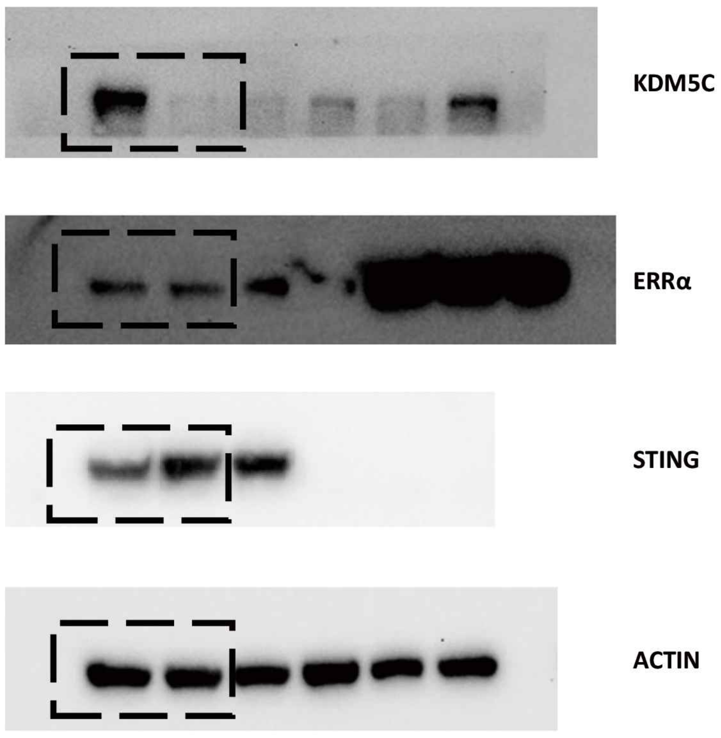

Figure S3E

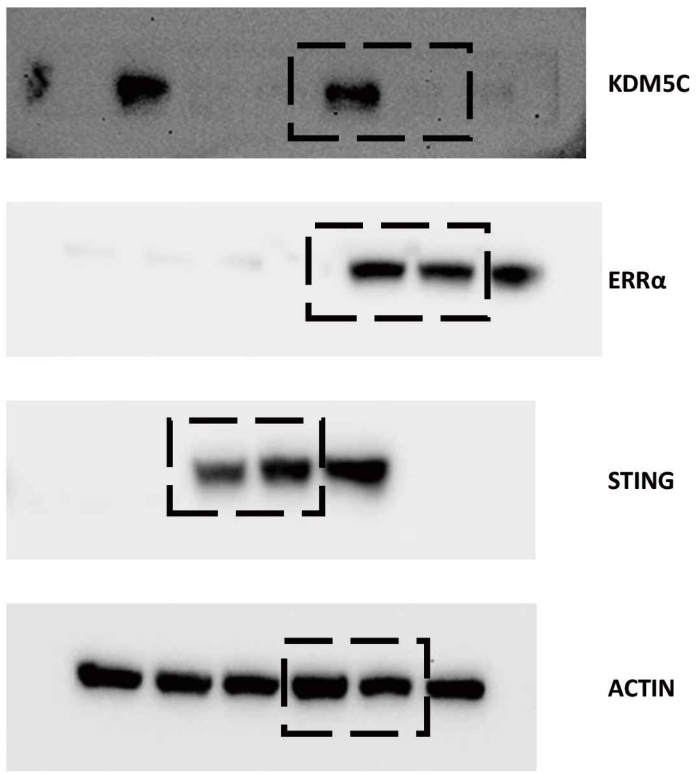

Figure S1B

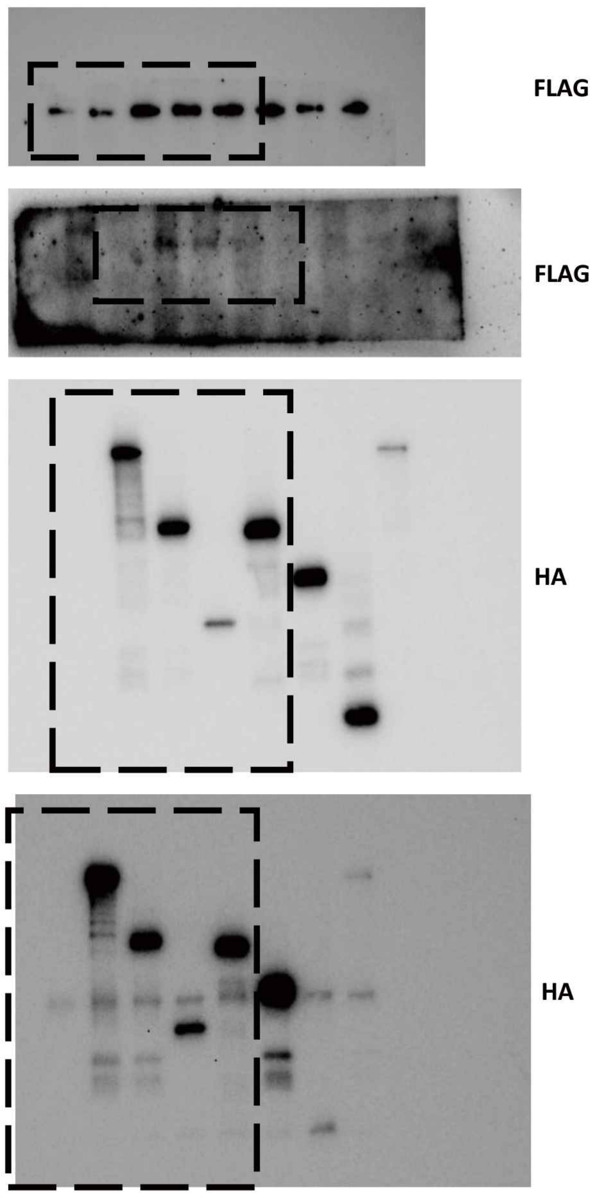

Figure S3F

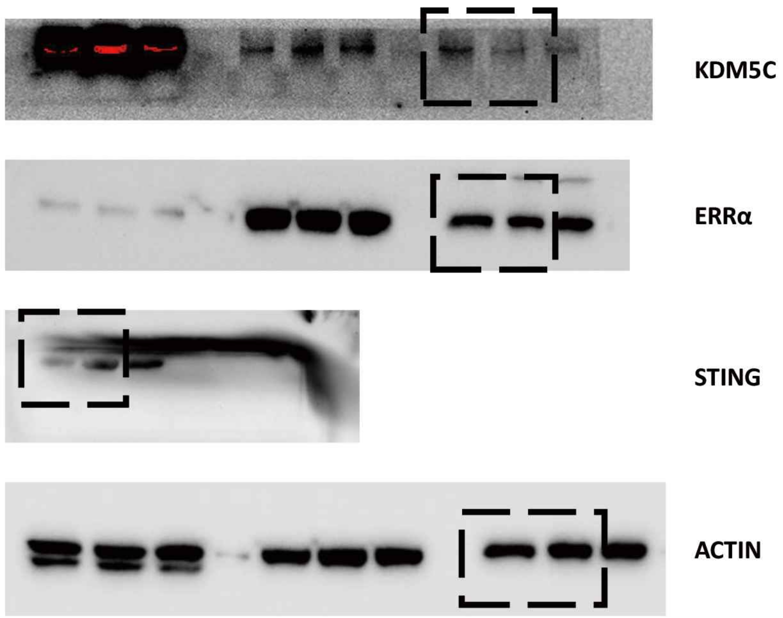

Figure S5C

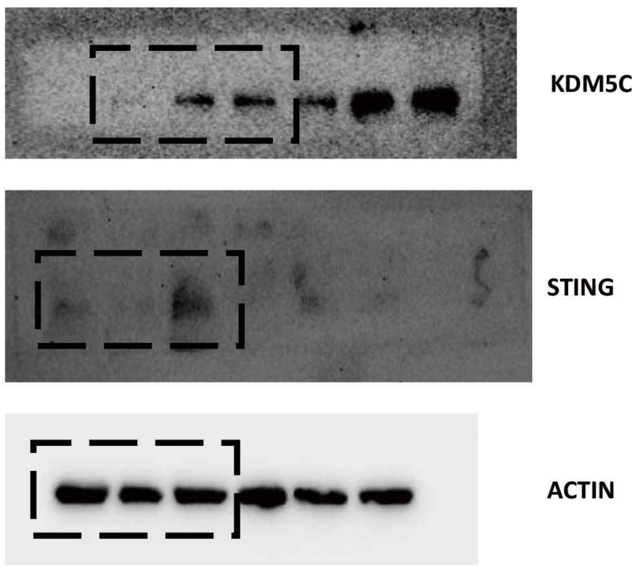

Figure S5D

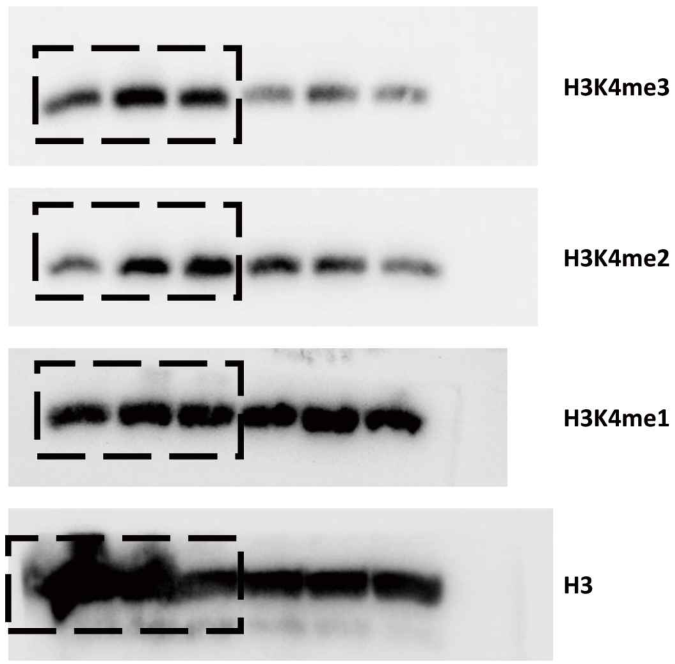

Figure S5E

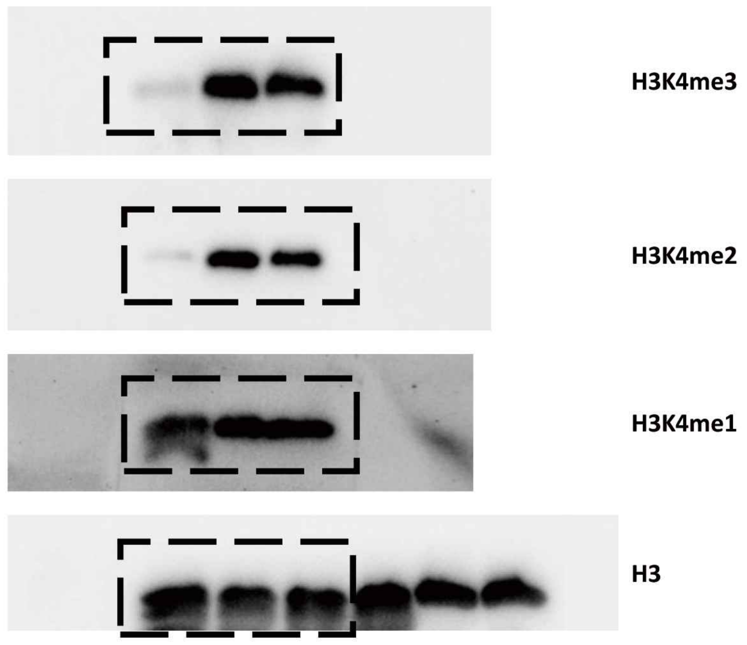

Supplement: Supplementary file 1 — Original Western blots [file 41419_2026_8499_MOESM1_ESM.pdf]
