# Supplementary material for: ERRα-KDM5C restrains STING enhancer activity to modulate type I interferon signaling in breast cancer progression
Source: Cell Death Dis. 2026 Feb 18;17(1):228. doi: 10.1038/s41419-026-08499-2 (PMC12920621; doi:10.1038/s41419-026-08499-2)
Supplement: Supplementary file 2 — Supplementary Figure Legends and Figures [file 41419_2026_8499_MOESM2_ESM.pdf]

SUPPLEMENTARY INFORMATION

SUPPLEMENTARY FIGURE LEGENDS

Figure S1. KDM5C Interacts with ERRα at Enhancers. Related to Figure 1.

(A) Cell extracts from HEK293T cells transfected with HA-tagged KDM5C and Flag-tagged ERRα were subjected to immunoprecipitation (IP) with anti-HA antibody followed by immunoblotting (IB) analysis as indicated.

(B) Cell extracts from HEK293T cells transfected with Flag-tagged ERRα and HA-tagged, full length (FL) or truncated forms of KDM5C were subjected to IP with anti-HA antibody followed by IB analysis as indicated. T1: aa (1–650); T2: aa (651–1000); T3: aa (1001–1560).

Figure S2. ERRα and KDM5C Repress Type I IFN-Stimulated Gene Expression. Related to Figure 2.

(A) Genome browser views of ESRRA (ERRα) gene based on RNA-seq are shown using the Integrative Genomics Viewer (IGV). Blue, shCTL; red, shERRα; purple, shKDM5C.

(B) Genome browser views of KDM5C gene based on RNA-seq are shown using the IGV. Blue, shCTL; red, shERRα; purple, shKDM5C.

(C) Enrichment scores from ssGSEA for selected Hallmark gene sets significantly enriched among genes negatively regulated by KDM5C or ERRα.

(D) Enrichment scores from ssGSEA for selected KEGG pathways significantly enriched among genes negatively regulated by KDM5C or ERRα.

Figure S3. ERRα and KDM5C Depletion Relieves STING Transcriptional Repression. Related to Figure 3.

(A-C) MCF7 **(A)**, MDA-MB-231 **(B)**, and HCC1937 cells **(C)** infected with shCTL or shKDM5C were subjected to RT-qPCR analysis and the expression of STING is shown. The experiments were repeated three times, and the representative data are shown (mean ± SEM; n = 3; $**P < 0.01$, $***P < 0.001$; unpaired Student's *t*-test, two-tailed).

(D-F) MCF7 **(D)**, MDA-MB-231 **(E)**, and HCC1937 cells **(F)** described in **(A-C)** were subjected to IB analysis using antibodies as indicated. Molecular weight is indicated on the left (in kDa).

Figure S4. Expression of Innate Immune Sensors after ERRα or KDM5C Knockdown. Related to Figure 3.

(A-C) Genomic views of mRNA expression levels and ERRα/KDM5C ChIP-seq signals at the promoters of cGAS **(A)**, MDA5 **(B)**, and MAVS **(C)** genes.

Figure S5. ERRα and KDM5C Depletion Increases STING Enhancer Activity. Related to Figure 4.

(A, B) MCF7 cells were infected with vectors expressing control vector, wild-type (WT) KDM5C, or catalytically inactive KDM5C (H514A). RT-qPCR analysis was performed to measure the expression levels of sense (+) **(A)** and antisense (-) **(B)** strand of STING enhancer RNA (eRNA). The experiments were repeated three times, and the representative data are shown (mean ± SEM; n = 3; $**P < 0.01$, $***P < 0.001$; unpaired Student's *t*-test, two-tailed).

(C) MCF7 cells were infected with the indicated vectors as in **(A, B)**. Whole-cell lysates were subjected to IB analysis with antibodies against KDM5C and β-actin (loading control). Molecular weight markers (in kDa) are indicated on the left.

(D, E) MCF7 cells were infected with lentivirus expressing control shRNA (shCTL) or two independent shRNAs targeting ERRα (shERRα-1, shERRα-2) **(D)** or KDM5C (shKDM5C-1, shKDM5C-2) **(E)**. Whole-cell lysates were subjected to IB analysis with antibodies against the indicated histone modifications: H3K4me3, H3K4me2, H3K4me1, and total Histone H3 (loading control). Molecular weight markers (in kDa) are shown on the left.

(F) Tag density profiles of H3K4me3 ChIP-seq signals in MCF7 cells at KDM5C and ERRα co-bound sites (± 3,000 bp) infected with control shRNA (shCTL) or two independent shRNAs targeting ERRα (shERRα-1, shERRα-2) or KDM5C (shKDM5C-1, shKDM5C-2).

(G) Box plot showing the H3K4me3 signal intensity at KDM5C and ERRα co-bound sites (± 500 bp) infected with control shRNA (shCTL) or two independent shRNAs targeting ERRα or KDM5C. The center line represents the median, the bounds of the box represent the interquartile range (IQR), and the whiskers extend to 1.5 × IQR (unpaired Student's *t*-test, two-tailed).

(H) Heat map depicting H3K4me3 ChIP-seq read density centered on KDM5C and ERRα co-bound sites (± 3,000 bp) infected with control shRNA or two independent shRNAs targeting ERRα or KDM5C.

Figure S6. ERRα and KDM5C Depletion Suppresses Breast Cancer Cell Growth via STING. Related to Figure 6.

(A) MCF7 cells were infected with shCTL or shKDM5C together with or without shSTING, followed by cell proliferation assay (n = 3; mean ± SEM; $^{***}P < 0.001$; day 4, by unpaired two-tailed Student's *t*-test).

(B) MCF7 cells were infected with shCTL or shKDM5C together with or without

shSTING, followed by colony formation assay.

(C) The quantification of the crystal violet dye in **(B)** is shown (n = 3; mean ± SEM; ***$P < 0.001$).

(D) MDA-MB-231 cells were infected with shCTL or shKDM5C together with or without shSTING, followed by cell proliferation assay (n = 3; mean ± SEM; ***$P < 0.001$; day 4, by unpaired two-tailed Student's *t*-test).

(E) MDA-MB-231 cells were infected with shCTL or shKDM5C together with or without shSTING, followed by colony formation assay.

(F) The quantification of the crystal violet dye in **(E)** is shown (n = 3; mean ± SEM; ***$P < 0.001$).

(G) HCC1937 cells were infected with shCTL or shKDM5C together with or without shSTING, followed by cell proliferation assay (n = 3; mean ± SEM; ***$P < 0.001$; day 4, by unpaired two-tailed Student's *t*-test).

(H) HCC1937 cells were infected with shCTL or shKDM5C together with or without shSTING, followed by colony formation assay.

(I) The quantification of the crystal violet dye in **(H)** is shown (n = 3; mean ± SEM; ***$P < 0.001$).

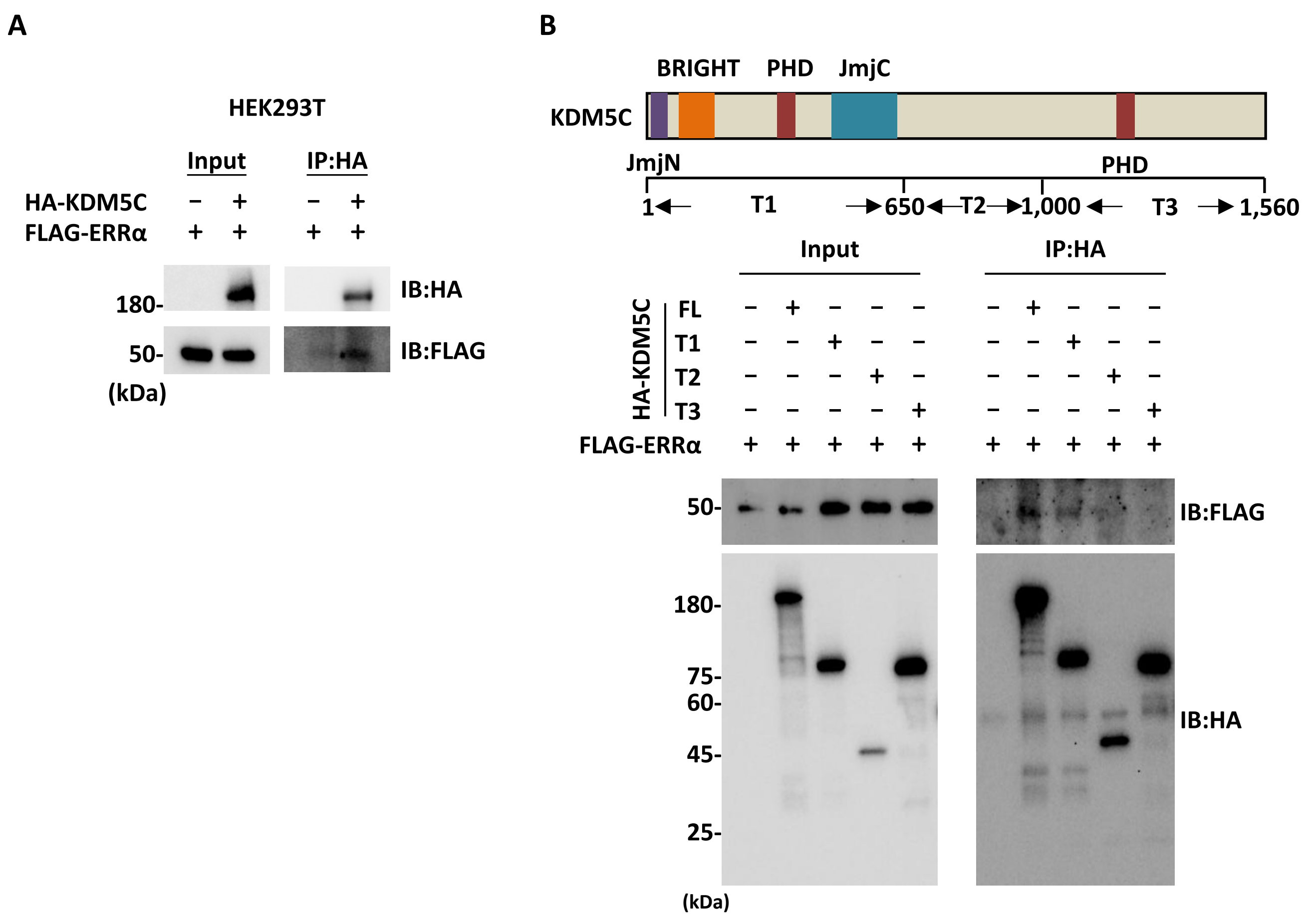
A
HEK293T
Input
IP:HA
HA-KDM5C
FLAG-ERRα
180-
50-
(kDa)
IB:HA
IB:FLAG
B
BRIGHT
PHD
JmjC
KDM5C
JmjN
PHD
1
T1
650
T2
1,000
T3
1,560
Input
IP:HA
HA-KDM5C
FL
T1
T2
T3
FLAG-ERRα
50-
180-
75-
60-
45-
25-
(kDa)
IB:FLAG
IB:HA

Figure S1

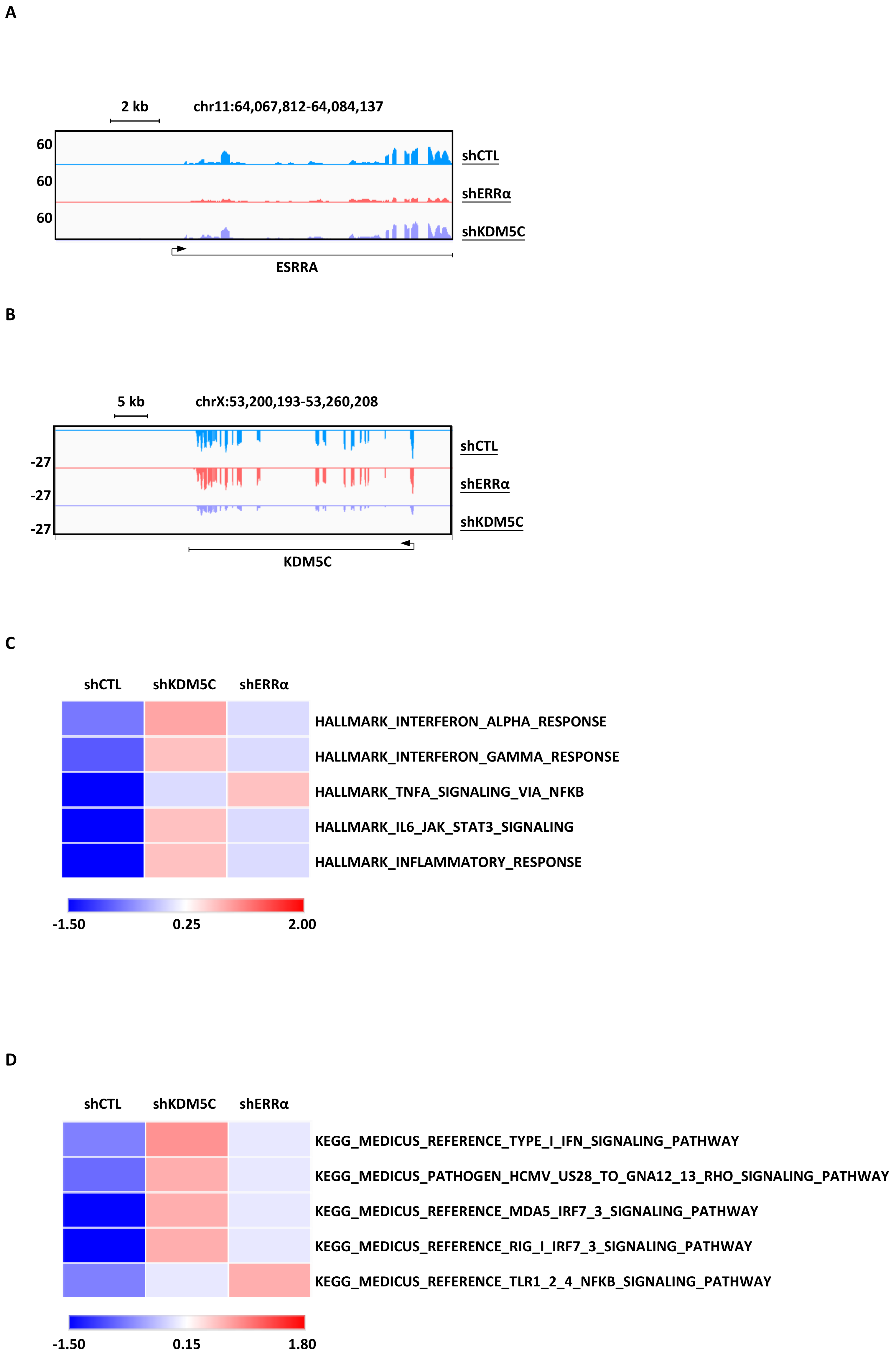
A
2 kb
chr11:64,067,812-64,084,137
60
60
60
shCTL
shERRα
shKDM5C
ESRRA
B
5 kb
chrX:53,200,193-53,260,208
-27
-27
-27
shCTL
shERRα
shKDM5C
KDM5C
C
shCTL
shKDM5C
shERRα
HALLMARK_INTERFERON_ALPHA_RESPONSE
HALLMARK_INTERFERON_GAMMA_RESPONSE
HALLMARK_TNFA_SIGNALING_VIA_NFKB
HALLMARK_IL6_JAK_STAT3_SIGNALING
HALLMARK_INFLAMMATORY_RESPONSE
-1.50
0.25
2.00
D
shCTL
shKDM5C
shERRα
KEGG_MEDICUS_REFERENCE_TYPE_I_IFN_SIGNALING_PATHWAY
KEGG_MEDICUS_PATHOGEN_HCMV_US28_TO_GNA12_13_RHO_SIGNALING_PATHWAY
KEGG_MEDICUS_REFERENCE_MDA5_IRF7_3_SIGNALING_PATHWAY
KEGG_MEDICUS_REFERENCE_RIG_I_IRF7_3_SIGNALING_PATHWAY
KEGG_MEDICUS_REFERENCE_TLR1_2_4_NFKB_SIGNALING_PATHWAY
-1.50
0.15
1.80

Figure S2

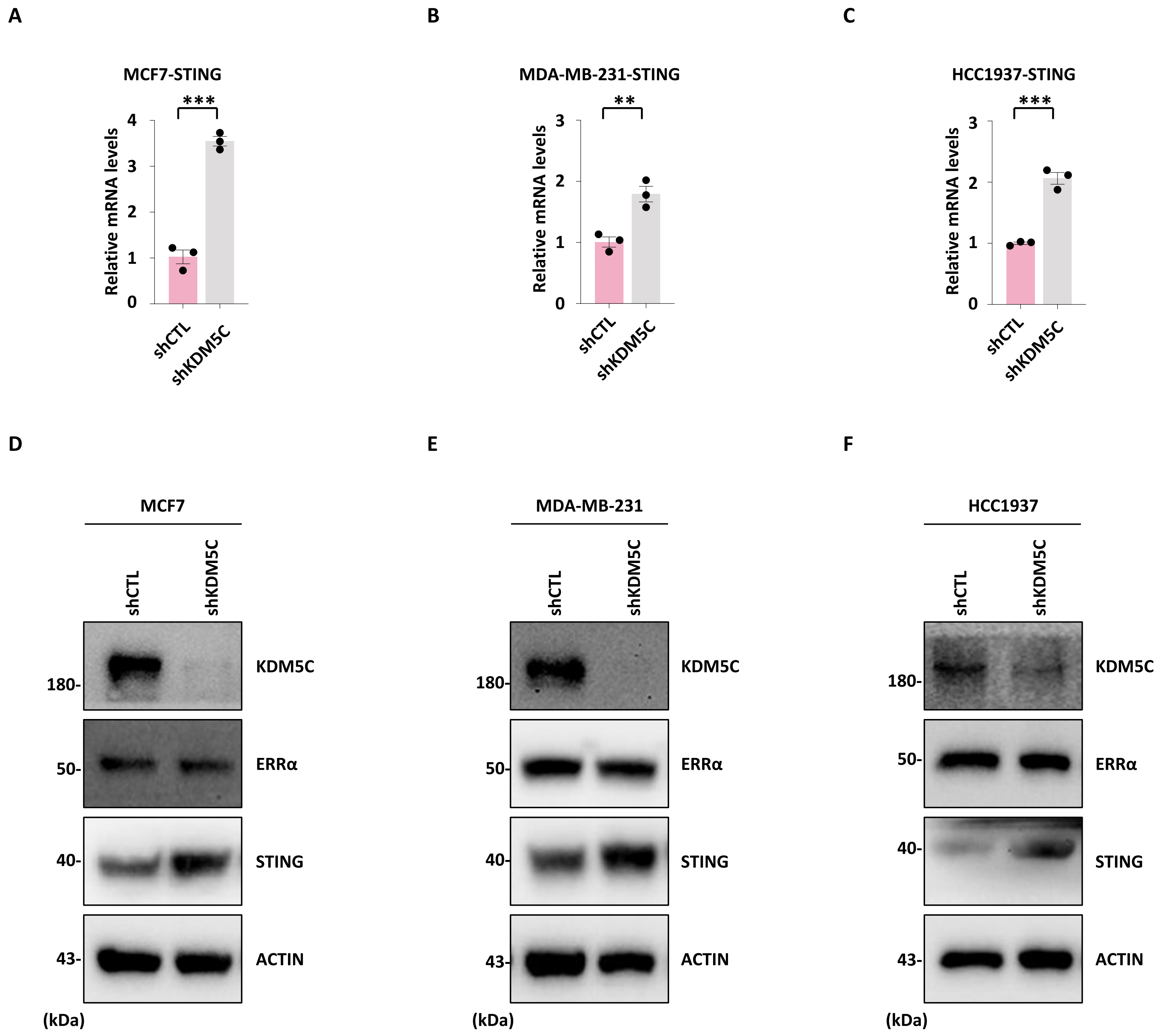
A
B
C
MCF7-STING
MDA-MB-231-STING
HCC1937-STING
***
**
***
Relative mRNA levels
shCTL
shKDM5C
D
E
F
MCF7
MDA-MB-231
HCC1937
KDM5C
ERRα
STING
ACTIN
180-
50-
40-
43-
(kDa)

Figure S3

A

B

C

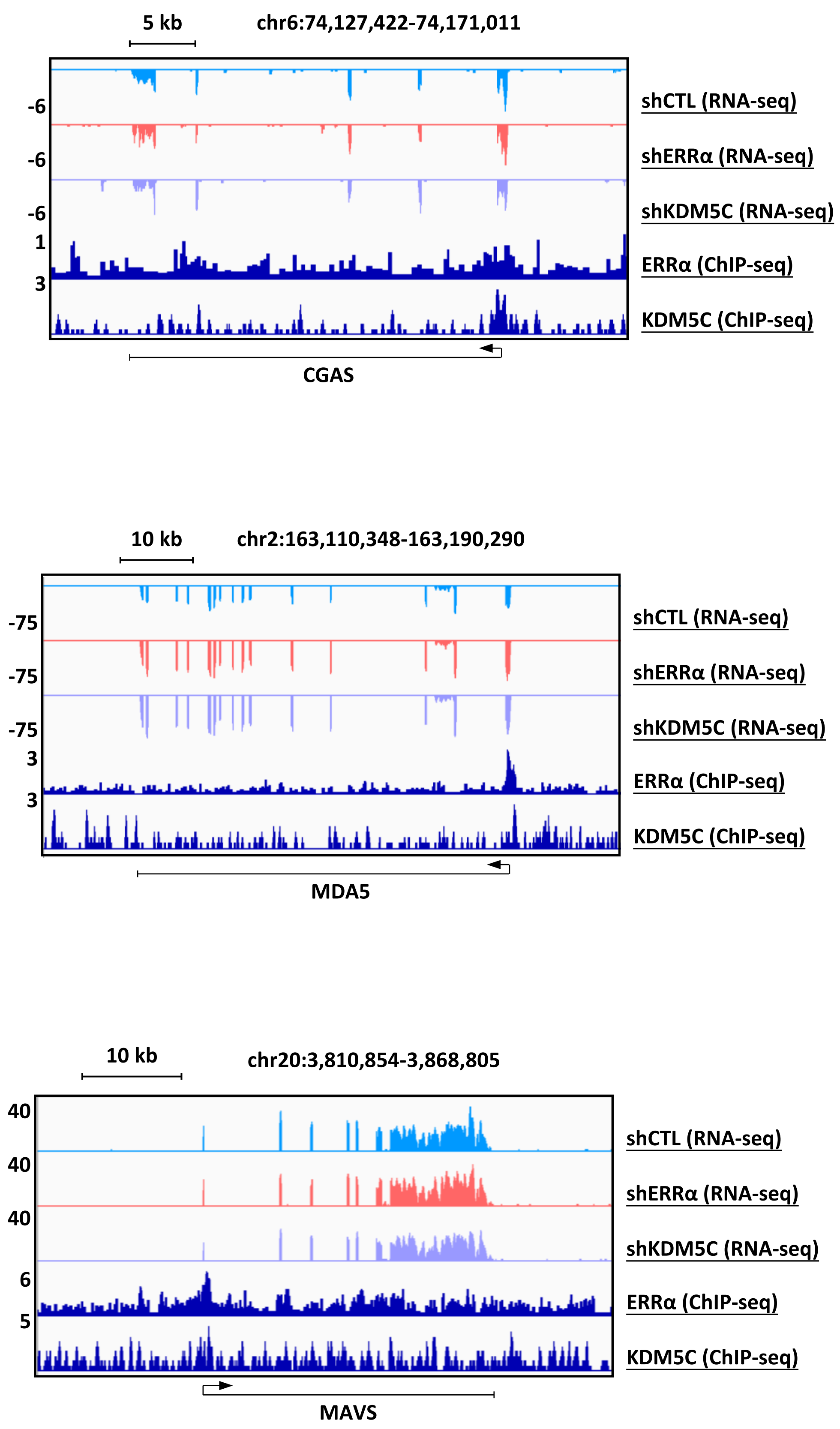

Figure S4

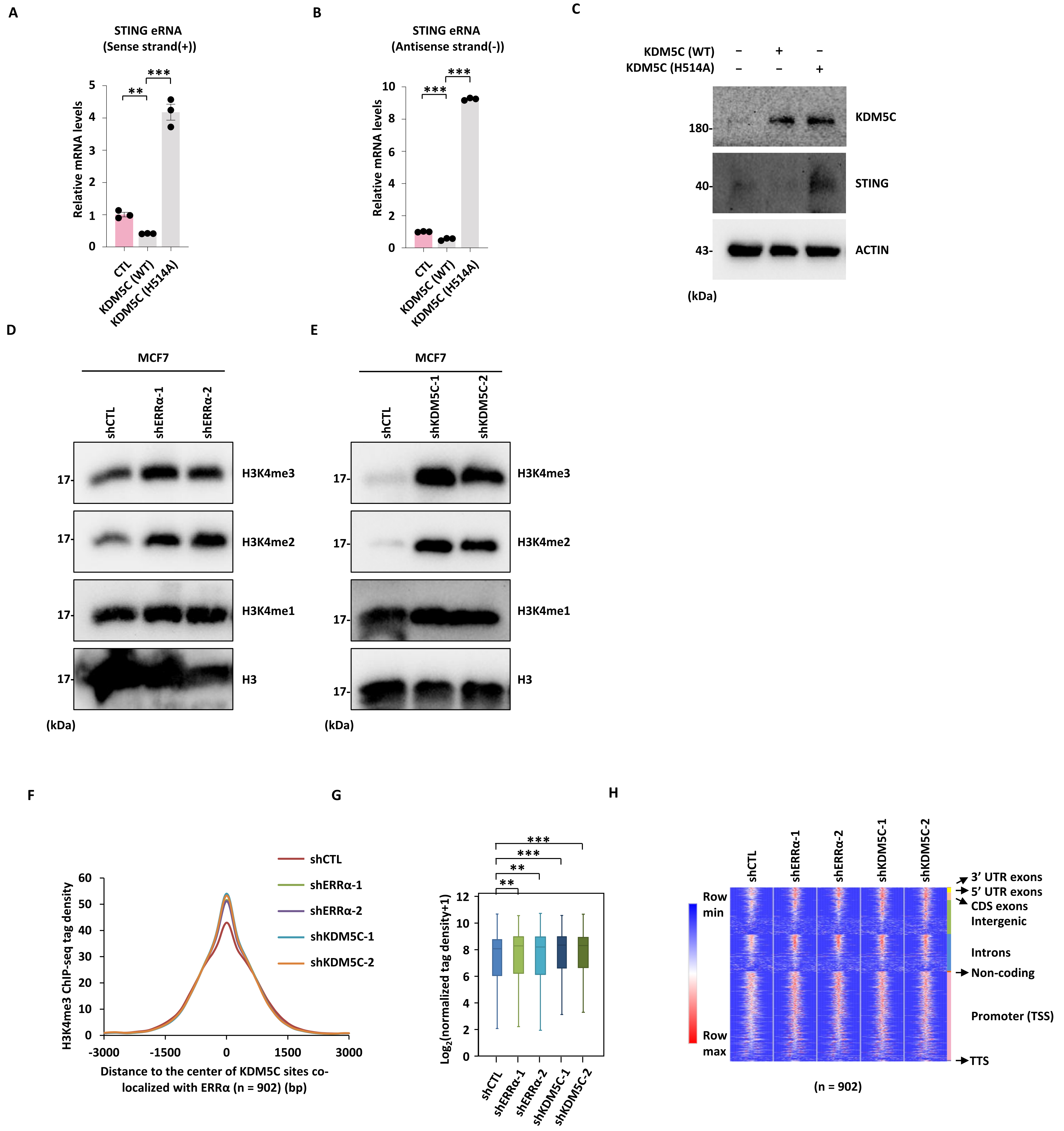
A
STING eRNA
(Sense strand(+))
Relative mRNA levels
CTL
KDM5C (WT)
KDM5C (H514A)
B
STING eRNA
(Antisense strand(-))
Relative mRNA levels
CTL
KDM5C (WT)
KDM5C (H514A)
C
KDM5C (WT) – + –
KDM5C (H514A) – – +
180-
KDM5C
40-
STING
43-
ACTIN
(kDa)
D
MCF7
shCTL
shERRα-1
shERRα-2
H3K4me3
H3K4me2
H3K4me1
H3
(kDa)
E
MCF7
shCTL
shKDM5C-1
shKDM5C-2
H3K4me3
H3K4me2
H3K4me1
H3
(kDa)
F
shCTL
shERRα-1
shERRα-2
shKDM5C-1
shKDM5C-2
H3K4me3 ChIP-seq tag density
Distance to the center of KDM5C sites co-localized with ERRα (n = 902) (bp)
G
Log2(normalized tag density+1)
shCTL
shERRα-1
shERRα-2
shKDM5C-1
shKDM5C-2
H
shCTL
shERRα-1
shERRα-2
shKDM5C-1
shKDM5C-2
Row min
Row max
3′ UTR exons
5′ UTR exons
CDS exons
Intergenic
Introns
Non-coding
Promoter (TSS)
TTS
(n = 902)

Figure S5

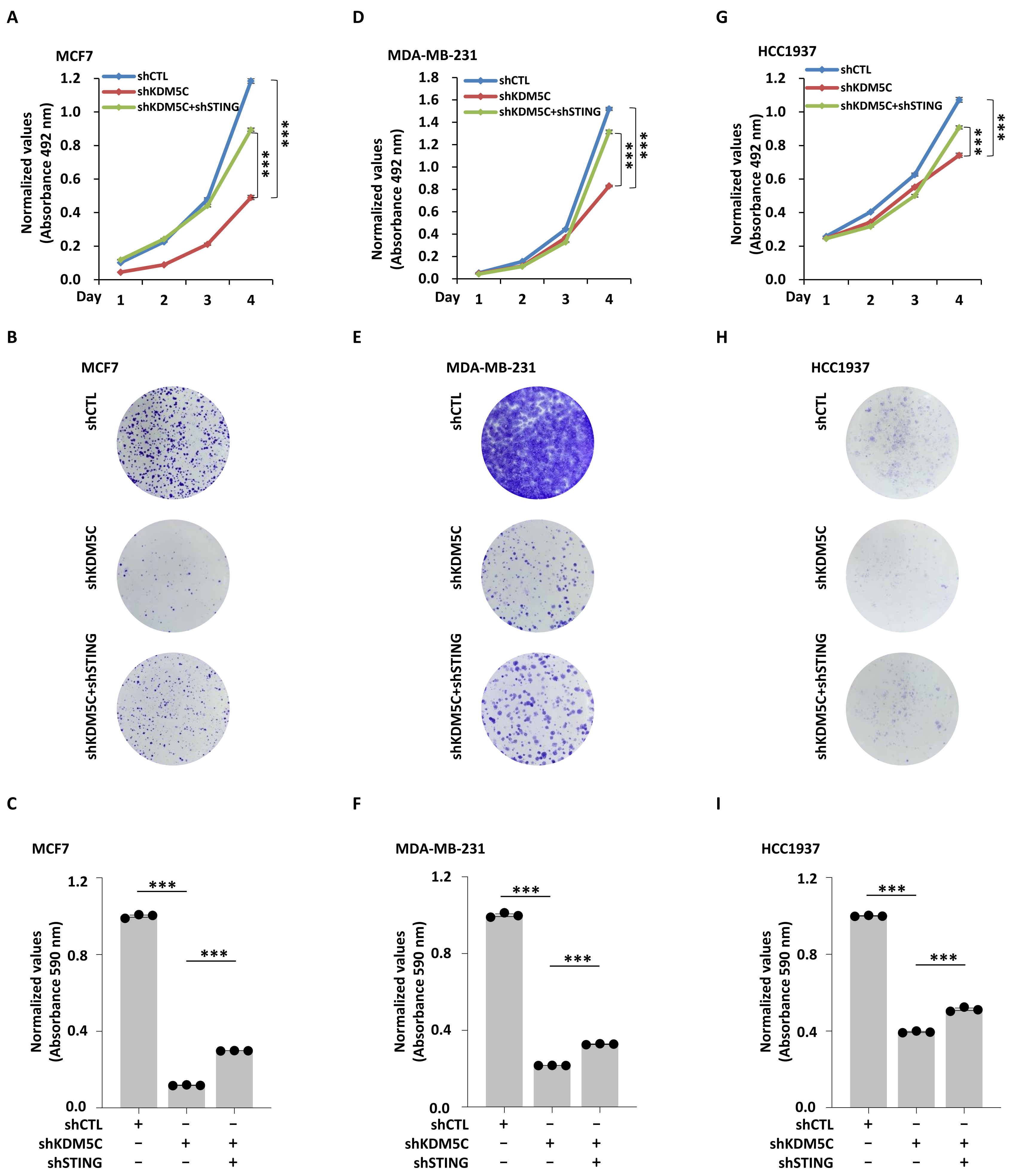
A
MCF7
shCTL
shKDM5C
shKDM5C+shSTING
Normalized values (Absorbance 492 nm)
Day
D
MDA-MB-231
G
HCC1937
B
E
H
shCTL
shKDM5C
shKDM5C+shSTING
C
F
I
Normalized values (Absorbance 590 nm)
shCTL
shKDM5C
shSTING

Figure S6
